# Sequence-Controlled Synthesis of Heteroaryl-Substituted Decahydroacridine-1,8-diones: Comparative Evaluation of Preformed Enaminone and Multicomponent Routes

**DOI:** 10.3390/molecules31152680

**Published:** 2026-07-31

**Authors:** Gökhan Özokan

**Affiliations:** Department of Chemistry, Faculty of Science, Yildiz Technical University, Davutpasa Campus, Istanbul 34220, Türkiye; gozokan@gmail.com

**Keywords:** acridine-1,8-dione, β-enaminone, dimedone, heteroaryl aldehyde, Michael addition, multicomponent reaction, reaction selectivity

## Abstract

Six 9-thienyl-10-aryl-substituted 3,3,6,6-tetramethyl-decahydroacridine-1,8-diones (**5a**–**f**) were prepared by two acid-mediated routes that used the same solvent, temperature, and reaction time. In the sequential route, preformed 5,5-dimethyl-3-(arylamino)cyclohex-2-enones were reacted with a heteroaryl aldehyde and dimedone; in the corresponding one-pot route, the arylamine, heteroaryl aldehyde, and two equivalents of dimedone were combined directly. The isolated yields of the product-forming second stage were 71–75%, corresponding to calculated overall two-step yields of 58.2–68.6% after accounting for the isolated enaminone-preparation yields. These overall yields remained higher than those of the one-pot procedure (40–47%), although the sequential protocol required an additional reaction, isolation, catalyst charge, and solvent-intensive operation. Structural assignments were based on the available IR, ^1^H NMR, ^13^C NMR, and EI-MS data. Tetrahydroacridinone by-products **6a**–**c** were isolated in 13–15% yields and showed diagnostic spectral changes relative to **5a**–**f**, including loss of the C-9 methine resonance and appearance of a strongly deshielded aromatic proton at 9.17–9.21 ppm. Within the limitations of an operational preparative comparison, the product distribution is consistent with a sequence-control interpretation in which preformation of the β-enaminone favors the 1:1:2 aldehyde/amine/dimedone product, whereas simultaneous component activation permits competing Knoevenagel, xanthene-forming, and aromatization pathways. This study provides a practical synthesis of thienyl-substituted decahydroacridine-1,8-diones and a mechanistically cautious explanation for the reduced selectivity of the one-pot process.

## 1. Introduction

Acridine-1,8-diones are conformationally constrained nitrogen heterocycles in which a central dihydropyridine-type ring is fused to two cyclic enone or cyclohexanone-derived units. The scaffold accommodates extensive substitution at C-9 and at the ring nitrogen, as illustrated by numerous crystallographically characterized derivatives [1,2,3,4,5,6,7,8,9,10]. Its electronic and conformational features have also supported applications as fluorescent and laser-active dyes [11], while acridine-related structures have been examined in medicinal-chemistry settings, including antiviral target classes [12]. Broader synthetic and biological aspects of acridine-1,8-diones have been reviewed recently [13].

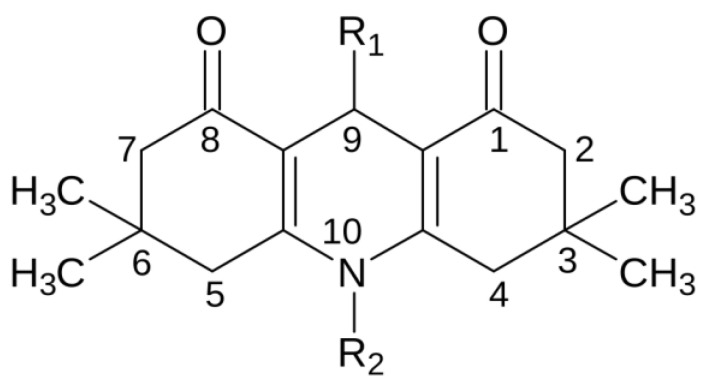



### 1.1. Synthetic Background and Study Rationale

The most widely used preparation is a Hantzsch-type pseudo-four-component condensation in which one aldehyde, two equivalents of dimedone and an ammonia or amine source undergo sequential Knoevenagel condensation, Michael addition and heteroannulation. Contemporary studies have focused mainly on improving this one-pot transformation through homogeneous, heterogeneous, recyclable or bio-based catalytic systems [14,15,16,17,18,19]. Although these methods can provide high yields, simultaneous generation of several nucleophilic and electrophilic intermediates can make the chemoselectivity strongly dependent on substrate structure, stoichiometry and reaction medium.

A complementary strategy employs an isolated 3-(arylamino)cyclohex-2-enone, or β-enaminone, as a preorganized nitrogen-containing building block. Reactions of such enaminones with aldehydes and 1,3-dicarbonyl compounds have been used to construct 9,10-disubstituted acridine-1,8-diones [20]. This sequence separates C-N bond formation from the carbon–carbon bond-forming stage and may, therefore, reduce competition between enaminone formation, aldehyde–dimedone condensation and oxygen-heterocycle formation.

Despite extensive catalyst-oriented work, comparatively less attention has been paid to a direct, internally controlled comparison of the preformed-enaminone and one-pot routes for heteroaryl aldehydes, or to the isolation of structurally informative by-products. Such information is important because isolated yield alone does not distinguish incomplete conversion from diversion of material into competing product manifolds. Thienyl aldehydes are particularly useful probes in this context: the heteroaryl unit is readily traced by NMR and EI-MS, and its retention or loss differentiates the principal product classes.

### 1.2. Objective and Scope

In this work, six 9-thienyl-10-aryl-decahydroacridine-1,8-diones **5a**–**f** were synthesized from three para-substituted arylamine-derived enaminones and two thiophene carbaldehydes. The sequential enaminone route and the corresponding multicomponent reaction were compared in the same *p*-toluenesulfonic acid/glacial acetic acid medium at the same temperature and reaction time. However, the archived protocols differed in substrate concentration and effective catalyst loading; the comparison is, therefore, an operational preparative comparison rather than a strictly isomolar kinetic study. The conclusions are restricted to the investigated acidic medium because no comparative solvent-control experiments were performed. The target products and tetrahydroacridinone by-products **6a**–**c** were assigned from the available IR, ^1^H NMR, ^13^C NMR, and nominal-mass EI-MS data. The proposed reaction network is presented as an evidence-based, nonexclusive connectivity model because the proposed intermediates were not monitored in situ, independently trapped, or established by kinetic experiments.

## 2. Materials and Methods

### 2.1. Materials and Instrumentation

Melting points were determined using a Gallenkamp apparatus; the original model and manufacturer details were not retained in the surviving records. ^1^H and ^13^C NMR spectra were recorded on a Varian Gemini spectrometer (Agilent Technologies Inc., Santa Clara, CA, USA) [400 MHz (^1^H) and 100 MHz (^13^C)], using tetramethylsilane (TMS) as the internal standard; chemical shifts are reported as δ values and coupling constants (*J*) in Hz. IR spectra were recorded on a PerkinElmer Spectrum One instrument (PerkinElmer Life and Analytical Sciences, Shelton, CT, USA) using KBr pellets. Archived mass spectra were acquired under electron-impact ionization; the original mass-spectrometer model was not retained in the surviving records. Reactions were monitored by TLC on silica gel plates (Merck 5554; Merck KGaA, Darmstadt, Germany) using ethyl acetate/hexane (1:1, *v*/*v*) as the eluent. Starting materials and reagents were obtained from commercial sources (Merck and Fluka) and used without further purification. Heteroaryl aldehydes were distilled before use.

#### Historical Data and Integrity Note

The synthetic work was carried out during the author’s post-doctoral research and in the subsequent continuation of that research approximately two decades ago. The numerical spectroscopic data were transcribed contemporaneously into the original laboratory records; they were not reconstructed during preparation of the present manuscript. A systematic search of the surviving post-doctoral and laboratory archives was undertaken, and all original instrument printouts that could be recovered are reproduced in the Supporting Information. The original printouts, raw electronic data, and residual samples for compounds **5b**, **5d**, **5e**, **6b**, and **6c** could not be recovered from the surviving post-doctoral and laboratory archives. No unavailable spectrum has been reconstructed, simulated, or presented as original experimental data.

Validation of the historical numerical records was documentary and internally comparative rather than a modern remeasurement. The transcribed values were checked against the surviving original printouts for related members of the same compound classes; molecular formulas, nominal molecular ions, and characteristic fragment losses; proton counts, multiplicities, coupling relationships, symmetry-equivalent carbon environments, and substituent-dependent 14 Da homologous shifts; the enaminone data in Ref. [21] and the related acridine-1,8-dione literature [1,2,3,4,5,6,7,8,9,10,20]; and consistency among the contemporaneous laboratory records, manuscript, and Supporting Information. These checks test chemical plausibility and identify transcriptional inconsistencies, but they are not equivalent to reacquiring spectra or reprocessing raw FID data.

### 2.2. General Procedure I: Synthesis of 5,5-Dimethyl-3-(arylamino)cyclohex-2-enones ***3a***–***c***

**Scheme 1:**
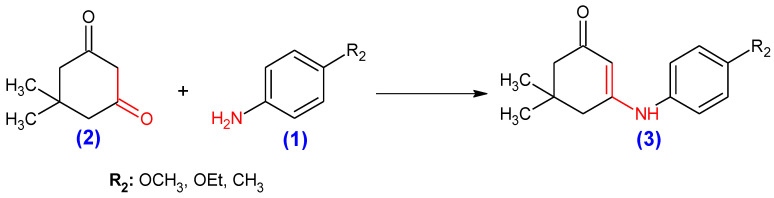
Compound identifiers: aromatic amines **1a**–**c**, dimedone **2**, and isolated enaminones **3a**–**c**.

5,5-Dimethyl-3-(arylamino)cyclohex-2-enones (**3a**–**c**) were synthesized according to a reported procedure [21] (Scheme 1).

A mixture of an aromatic amine (**1a**–**c**, 1.2 mmol), dimedone (**2**, 1.0 mmol) and *p*-toluenesulfonic acid (0.08 mmol) in benzene (10 mL) was refluxed for 4 h. The solvent was removed under reduced pressure, and the crude product was purified by recrystallization from ethyl acetate.

5,5-Dimethyl-3-[(4-methoxyphenyl)amino]cyclohex-2-en-1-one (3a, C_15_H_19_NO_2_)

This compound was obtained by following the above general procedure (I), by refluxing **1a** and **2** in 94% (lit. [21]: 66%) yield as yellow crystals, mp 119–120 °C; ^1^H-NMR (CDCl_3_) δ: 1.07 (s, 6H, 2 × CH_3_), 2.17 (s, 2H, 4-H), 2.30 (s, 2H, 6-H), 3.78 (s, 3H, OCH_3_), 5.35 (s, 1H, 2-H), 6.58 (br.s, 1H, NH), 6.83 (d, 2H, 2′- and 6′-H, ^3^*J*_2′,3′_ = ^3^*J*_5′,6′_ = 9.0 Hz), 7.05 (d, 2H, 3′- and 5′-H) [lit. [21]: 1.10 (s, 6H), 2.20 (s, 2H), 2.33 (s, 2H), 3.80 (s, 3H), 5.38 (s, 1H), 6.59 (br.s, 1H), 6.85 (d, 2H), 7.07 (d, 2H)]; ^13^C-NMR (CDCl_3_) δ: 28.54 (2 × CH_3_), 33.07 (C5), 43.52 (C4), 50.51 (C6), 55.73 (OCH_3_), 97.97 (C2), 114.72 (C3′ and C5′), 126.46 (C2′ and C6′), 131.04 (C1′), 157.90 (C4′), 162.15 (C3), 197.76 (C1) [lit. [21]: 28.6 (2C), 33.1, 43.5, 50.5, 55.7, 98.0, 114.7 (2C), 126.5 (2C), 131.0, 157.9, 162.2, 197.7]; IR (KBr) νmax/cm^−1^: 3206 (N-H), 3009 (aromatic, =CH), 2955, 2901 and 2831 (CH_3_), 1607 and 1537 (C=C-C=O); EIMS *m*/*z*: 246 [M + 1, 17%], 245 [M+, 100%], 230 [M+ –(CH_3_), 11%], 217 [M+ –(CO), 10%], 202 [M+ –(CH_2_-CO), 11%], 189 [M+ –(C_4_H_8_), 85%], 174 [M+ –(C_5_H_10_), 63%], 160 [M+ –(C_5_H_8_O), 11%] [lit. [21]: 246 (M + 1)].

5,5-Dimethyl-3-[(4-ethoxyphenyl)amino]cyclohex-2-en-1-one (3b, C_16_H_21_NO_2_)

This compound was obtained by following the above general procedure (I), by refluxing **1b** and **2** in 85% yield as yellow crystals, mp 128–130 °C; ^1^H-NMR (CDCl_3_) δ: 1.03 (s, 6H, 2 × CH_3_), 1.39 (t, 3H, OCH_2_CH_3_), 2.15 (s, 2H, 4-H), 2.31 (s, 2H, 6-H), 3.98 (q, 2H, OCH_2_CH_3_), 5.38 (s, 1H, 2-H), 6.79 (d, 2H, 2′- and 6′-H, *^3^J_2_*_′,3′_ = ^3^*J*_5′,6′_ = 8.8 Hz), 7.03 (d, 2H, 3′- and 5′-H), 7.35 (br.s, 1H, NH); ^13^C-NMR (CDCl_3_) δ: 15.01 (OCH_2_**C**H_3_), 28.45 (2 × CH_3_), 33.03 (C5), 43.24 (C4), 49.98 (C6), 63.94 (O**C**H_2_CH_3_), 97.37 (C2), 115.22 (C3′ and C5′), 126.37 (C2′ and C6′), 130.79 (C1′), 157.30 (C4′), 163.73 (C3), 197.22 (C1); IR (KBr) νmax/cm^−1^: 3245 (N-H), 3048 (aromatic, =CH), 2977, 2956 and 2868 (CH_3_), 1574 and 1537 (C=C-C=O); EIMS *m*/*z*: 260 [M + 1, 18%], 259 [M+, 100%], 244 [M+ –(CH_3_), 9%], 230 [M+ –(CO), 14%], 216 [M+ –(CH_2_-CO), 12%], 203 [M+ –(C_4_H_8_), 52%], 174 [M+ –(C_5_H_8_O), 58%].

5,5-Dimethyl-3-[(4-methylphenyl)amino]cyclohex-2-en-1-one (3c, C_15_H_19_NO)

This compound was obtained by following the above general procedure (I), by refluxing **1c** and **2** in 82% (lit. [21]: 57%) yield as yellow crystals, mp 200–203 °C (lit. [21]: mp 203–204 °C); ^1^H-NMR (CDCl_3_) δ: 1.05 (s, 6H, 2 × CH_3_), 2.16 (s, 2H, 4-H), 2.30 (s, 3H, *p*-tolyl CH_3_), 2.32 (s, 2H, 6-H), 5.48 (s, 1H, 2-H), 7.00 (d, 2H, 2′- and 6′-H, ^3^*J*_2′,3′_ = ^3^*J*_5′,6′_ = 8.2 Hz), 7.08 (d, 2H, 3′- and 5′-H), 7.08 (br.s, 1H, NH) [lit. [21]: 1.06 (s, 6H), 2.18 (s, 2H), 2.33 (s, 2H), 5.49 (s, 1H), 7.01 (d, 2H), 7.09 (d, 2H), 7.22 (br.s, 1H)]; 13C-NMR (CDCl3) δ: 21.17 (*p*-tolyl CH3), 28.51 (2 × CH_3_), 33.02 (C5), 43.55 (C4), 50.51 (C6), 98.06 (C2), 124.28 (C2′ and C6′), 130.02 (C3′ and C5′), 135.57 and 135.82 (C1′ and C4′), 161.78 (C3), 197.98 (C1) [lit. [21]: 28.5 (2C), 33.0, 43.5, 50.6, 98.0, 124.3 (2C), 130.0 (2C), 135.5, 135.9, 161.9, 198.0]; IR (KBr) νmax/cm^−1^: 3243 (N-H), 3059 (aromatic, =CH), 2956, 2920 and 2884 (CH_3_), 1607 and 1575 (C=C-C=O); EIMS *m*/*z*: 230 [M + 1, 12%], 229 [M+, 62%], 214 [M+ –(CH_3_), 10%], 201 [M+ –(CO), 8%], 186 [M+ –(CH_2_-CO), 12%], 173 [M+ –(C_4_H_8_), 100%], 158 [M+ –(C_5_H_10_), 7%], 144 [M+ –(C_5_H_8_O), 28%] [lit. [21]: 230 (M + 1)].

### 2.3. General Procedure II: Sequential Enaminone Route to ***5a***–***f***

**Scheme 2:**
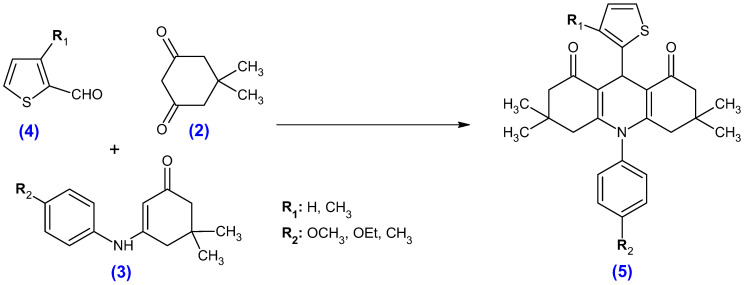
Sequential route: enaminones **3a**–**c**, heteroaryl aldehydes **4a**,**b**, dimedone **2**, and target products **5a**–**f**.

A mixture of 5,5-dimethyl-3-(arylamino)cyclohex-2-enone (**3a**–**c**, 1.0 mmol), heteroaryl aldehyde (**4a**,**b**, 1.0 mmol), dimedone (**2**, 1.0 mmol) and *p*-toluenesulfonic acid (0.08 mmol) in glacial acetic acid (5 mL) was refluxed for 4 h. Ethanol (10 mL) was added, and the solvent was removed under reduced pressure. The oily residue was dissolved in diethyl ether/ethanol (20:1), washed with saturated Na_2_CO_3_ solution (3 × 20 mL) and water (3 × 20 mL), and dried over anhydrous Na_2_SO_4_. The solvent was evaporated under reduced pressure, and the crude product was purified by recrystallization from diethyl ether/ethanol (Scheme 2).

### 2.4. General Procedure III: Multicomponent Route to ***5a***–***f***

**Scheme 3:**
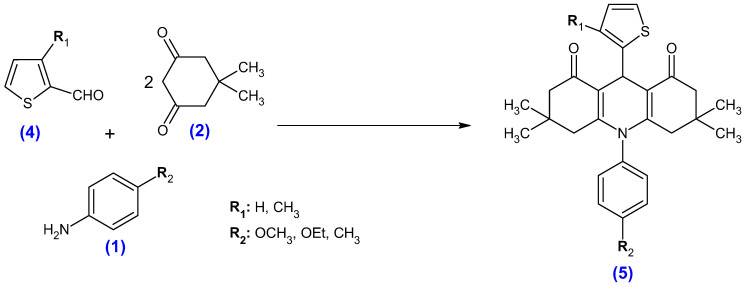
Multicomponent route: aromatic amines **1a**–**c**, heteroaryl aldehydes **4a**,**b**, dimedone **2**, and target products **5a**–**f**.

A mixture of aromatic amine (**1a**–**c**, 0.5 mmol), heteroaryl aldehyde (**4a**,**b**, 0.5 mmol), dimedone (**2**, 1.0 mmol) and *p*-toluenesulfonic acid (0.08 mmol) in glacial acetic acid (5 mL) was refluxed for 4 h. Ethanol (10 mL) was added, and the solvent was removed under reduced pressure. The oily residue was dissolved in diethyl ether/ethanol (20:1), washed with saturated Na_2_CO_3_ solution (3 × 20 mL) and water (3 × 20 mL), and dried over anhydrous Na_2_SO_4_. The solvent was evaporated under reduced pressure, and the crude product was purified by recrystallization from diethyl ether/ethanol (Scheme 3).

#### Nominal Concentration and Catalyst-Loading Comparison

In procedure II, enaminone **3**, aldehyde **4**, and dimedone **2** were each 0.200 M (1.0 mmol in 5 mL), while *p*-toluenesulfonic acid was 0.016 M, corresponding to 8 mol% relative to each 1.0 mmol substrate; the total nominal substrate concentration was 0.600 M. In procedure III, the amine and aldehyde were each 0.100 M (0.5 mmol in 5 mL), dimedone was 0.200 M, and *p*-toluenesulfonic acid was 0.016 M, corresponding to 16 mol% relative to the limiting amine/aldehyde and 8 mol% relative to dimedone; the total nominal substrate concentration was 0.400 M. These differences preclude unambiguous attribution of the yield difference solely to the order of component addition or to sequence-dependent kinetics.

The numerical characterization data below were transcribed contemporaneously from the original laboratory records. The Supporting Information reproduces every original archived spectrum that could be recovered and clearly identifies the compounds for which only historical numerical records survive. The assigned proton count is reported for every ^1^H NMR resonance. Reliable numerical integral traces and raw FID files could not be recovered from the surviving post-doctoral and laboratory archives and were, therefore, not reconstructed retrospectively.

9-(2-Thienyl)-10-(4-methoxyphenyl)-3,3,6,6-tetramethyl-1,8-dioxo-1,2,3,4,5,6,7,8,9,10-decahydroacridine (5a, C_28_H_31_NO_3_S)

This compound was obtained by following the above general procedure (II), by refluxing **3a**, **4a** and **2** in 73% yield as colorless crystals, mp 244 °C; ^1^H-NMR (CDCl_3_) δ: 0.85 (s, 6H, 2 × CH_3_), 0.95 (s, 6H, 2 × CH_3_), 1.83 (d, 2H, 4- and 5-H, ^2^*J*_4,4′_ = ^2^*J*_5,5′_ = 17.6 Hz), 2.08 (d, 2H, 4′- and 5′-H), 2.18 (d, 2H, 2- and 7-H, ^2^*J*_2,2′_ = ^2^*J*_7,7′_ = 16.5 Hz), 2.21 (d, 2H, 2′- and 7′-H), 3.90 (s, 3H, OCH_3_), 5.65 (s, 1H, 9-H), 6.83–7.19 (m, 7H, hetaryl and aryl); ^13^C-NMR (CDCl_3_) δ: 26.99 (2 × CH_3_), 27.51, 30.14, 32.55, 41.86 (C4 and C5), 50.45 (C2 and C7), 55.83 (OCH_3_), 114.35–150.81 (C1a, C8a, hetaryl and aryl), 160.06 (C4a and C5a), 195.88 (C1 and C8); IR (KBr) νmax/cm^−1^: 3064 (aromatic, =CH), 2959 and 2870 (CH_3_), 1634 (C=O), 1574 (C=C), 1512 and 1464 (aromatic, C=C); EIMS *m*/*z*: 462 [M + 1, 33%], 461 [M+, 100%], 446 [M+ –(CH_3_), 4%], 433 [M+ –(CO), 5%], 378 [M+ –(thienyl), 87%].

9-(2-Thienyl)-10-(4-ethoxyphenyl)-3,3,6,6-tetramethyl-1,8-dioxo-1,2,3,4,5,6,7,8,9,10-decahydroacridine (5b, C_29_H_33_NO_3_S)

This compound was obtained by following the above general procedure (II), by refluxing **3b**, **4a** and **2** in 75% yield as colorless crystals, mp 204–206 °C; ^1^H-NMR (CDCl_3_) δ: 0.85 (s, 6H, 2 × CH_3_), 0.95 (s, 6H, 2 × CH_3_), 1.43 (t, 3H, OCH_2_CH_3_), 1.83 (d, 2H, 4- and 5-H, ^2^*J*_4,4′_ = ^2^*J*_5,5′_ = 17.6 Hz), 2.08 (d, 2H, 4′- and 5′-H), 2.18 (d, 2H, 2- and 7-H, ^2^*J*_2,2′_ = ^2^*J*_7,7′_ = 16.5 Hz), 2.22 (d, 2H, 2′- and 7′-H), 4.05 (q, 2H, OCH_2_CH_3_), 5.65 (s, 1H, 9-H), 6.83–7.18 (m, 7H, hetaryl and aryl); ^13^C-NMR (CDCl_3_) δ: 15.02 (OCH_2_**C**H_3_), 26.99 (2 × CH_3_), 27.52, 30.10, 32.56, 41.85 (C4 and C5), 50.46 (C2 and C7), 63.98 (O**C**H_2_CH_3_), 114.36–149.12 (C1a, C8a, hetaryl and aryl), 160.00 (C4a and C5a), 195.77 (C1 and C8); IR (KBr) νmax/cm^−1^: 3064 (aromatic, =CH), 2959 and 2870 (CH_3_), 1634 (C=O), 1574 (C=C), 1512 and 1464 (aromatic, C=C); EIMS *m*/*z*: 476 [M + 1, 35%], 475 [M+, 100%], 460 [M+ –(CH_3_), 5%], 447 [M+ –(CO), 5%], 392 [M+ –(thienyl), 88%].

9-(2-Thienyl)-10-(4-methylphenyl)-3,3,6,6-tetramethyl-1,8-dioxo-1,2,3,4,5,6,7,8,9,10-decahydroacridine (5c, C_28_H_31_NO_2_S)

This compound was obtained by following the above general procedure (II), by refluxing **3c**, **4a** and **2** in 75% yield as colorless crystals, mp 123–125 °C; ^1^H-NMR (CDCl_3_) δ: 0.86 (s, 6H, 2 × CH_3_), 0.95 (s, 6H, 2 × CH_3_), 1.82 (d, 2H, 4- and 5-H, ^2^*J*_4,4′_ = ^2^*J*_5,5′_ = 17.6 Hz), 2.08 (d, 2H, 4′- and 5′-H), 2.19 (d, 2H, 2- and 7-H, ^2^*J*_2,2′_ = ^2^*J*_7,7′_ = 16.5 Hz), 2.22 (d, 2H, 2′- and 7′-H), 2.47 (s, 3H, *p*-tolyl CH_3_), 5.66 (s, 1H, 9-H), 6.84–7.11 (m, 7H, hetaryl and aryl); ^13^C-NMR (CDCl_3_) δ: 21.52 (*p*-tolyl CH_3_), 26.96 (2 × CH_3_), 27.56, 30.09, 32.58, 41.86 (C4 and C5), 50.45 (C2 and C7), 114.34–139.75 (C1a, C8a, hetaryl and aryl), 150.58 (C4a and C5a), 195.97 (C1 and C8); IR (KBr) νmax/cm^−1^: 3067 (aromatic, =CH), 2957 and 2868 (CH_3_), 1645 (C=O), 1573 (C=C), 1510 and 1454 (aromatic, C=C); EIMS *m*/*z*: 446 [M + 1, 35%], 445 [M+, 100%], 430 [M+ –(CH_3_), 5%], 417 [M+ –(CO), 5%], 362 [M+ –(thienyl), 97%].

9-(3-Methyl-2-thienyl)-10-(4-methoxyphenyl)-3,3,6,6-tetramethyl-1,8-dioxo-1,2,3,4,5,6,7,8,9,10-decahydroacridine (5d, C_29_H_33_NO_3_S)

This compound was obtained by following the above general procedure (II), by refluxing **3a**, **4b** and **2** in 73% yield as colorless crystals, mp 269–271 °C; ^1^H-NMR (CDCl_3_) δ: 0.85 (s, 6H, 2 × CH_3_), 0.94 (s, 6H, 2 × CH_3_), 1.82 (d, 2H, 4- and 5-H, ^2^*J_4_*_,4′_ = ^2^*J*_5,5′_ = 17.4 Hz), 2.07 (d, 2H, 4′- and 5′-H), 2.15 (d, 2H, 2- and 7-H, *^2^J_2_*_,2′_ = ^2^*J*_7,7′_ = 15.9 Hz), 2.19 (d, 2H, 2′- and 7′-H), 2.48 (s, 3H, thienyl-CH_3_), 3.84 (s, 3H, OCH_3_), 5.55 (s, 1H, 9-H), 6.64 (d, 1H, thienyl 4″-H, ^3^*J*_4″, 5″_ = 5.1 Hz), 6.89 (d, 1H, thienyl 5″-H), 7.08–7.33 (m, 4H, aryl); ^13^C-NMR (CDCl_3_) δ: 14.29 (thienyl-CH_3_), 26.92 (2 × CH_3_), 30.03, 30.05, 32.58, 41.90 (C4 and C5), 50.46 (C2 and C7), 55.75 (OCH_3_), 115.38–145.00 (C1a, C8a, hetaryl and aryl), 149.85 (C4a and C5a), 195.81 (C1 and C8); IR (KBr) νmax/cm^−1^: 3055 (aromatic, =CH), 2952 and 2865 (CH_3_), 1641 (C=O), 1571 (C=C), 1510 and 1422 (aromatic, C=C); EIMS *m*/*z*: 476 [M + 1, 6%], 475 [M+, 15%], 460 [M+ –(CH_3_), 11%], 446 [M+ –(CO), 3%], 378 [M+ –(thienyl-CH_3_), 100%].

9-(3-Methyl-2-thienyl)-10-(4-ethoxyphenyl)-3,3,6,6-tetramethyl-1,8-dioxo-1,2,3,4,5,6,7,8,9,10-decahydroacridine (5e, C_30_H_35_NO_3_S)

This compound was obtained by following the above general procedure (II), by refluxing **3b**, **4b** and **2** in 72% yield as colorless crystals, mp 229–232 °C; ^1^H-NMR (CDCl_3_) δ: 0.85 (s, 6H, 2 × CH_3_), 0.94 (s, 6H, 2 × CH_3_), 1.43 (t, 3H, OCH_2_CH_3_), 1.83 (d, 2H, 4- and 5-H, ^2^*J*_4,4′_ = ^2^*J*_5,5′_ = 17.4 Hz), 2.07 (d, 2H, 4′- and 5′-H), 2.14 (d, 2H, 2- and 7-H, ^2^*J*_2,2′_ = ^2^*J*_7,7′_ = 15.9 Hz), 2.18 (d, 2H, 2′- and 7′-H), 2.48 (s, 3H, thienyl-CH_3_), 4.05 (q, 2H, OCH_2_CH_3_), 5.53 (s, 1H, 9-H), 6.64 (d, 1H, thienyl 4″-H, ^3^*J*_4″, 5″_ = 5.1 Hz), 6.89 (d, 1H, thienyl 5″-H), 7.08–7.30 (m, 4H, aryl); ^13^C-NMR (CDCl_3_) δ: 14.31 (thienyl-CH_3_), 15.05 (OCH_2_**C**H_3_), 26.90 (2 × CH_3_), 30.06, 30.07, 32.58, 41.92 (C4 and C5), 50.44 (C2 and C7), 64.00 (O**C**H_2_CH_3_), 115.38–144.05 (C1a, C8a, hetaryl and aryl), 149.82 (C4a and C5a), 195.85 (C1 and C8); IR (KBr) νmax/cm^−1^: 3053 (aromatic, =CH), 2950 and 2866 (CH_3_), 1640 (C=O), 1572 (C=C), 1511 and 1423 (aromatic, C=C); EIMS *m*/*z*: 490 [M + 1, 5%], 489 [M+, 15%], 474 [M+ –(CH_3_), 11%], 460 [M+ –(CO), 3%], 392 [M+ –(thienyl-CH_3_), 100%].

9-(3-Methyl-2-thienyl)-10-(4-methylphenyl)-3,3,6,6-tetramethyl-1,8-dioxo-1,2,3,4,5,6,7,8,9,10-decahydroacridine (5f, C_29_H_33_NO_2_S)

This compound was obtained by following the above general procedure (II), by refluxing **3c**, **4b** and **2** in 71% yield as colorless crystals, mp 279–282 °C; ^1^H-NMR (CDCl_3_) δ: 0.84 (s, 6H, 2 × CH_3_), 0.94 (s, 6H, 2 × CH_3_), 1.82 (d, 2H, 4- and 5-H, ^2^*J*_4,4′_ = ^2^*J*_5,5′_ = 17.4 Hz), 2.06 (d, 2H, 4′- and 5′-H), 2.14 (d, 2H, 2- and 7-H, ^2^*J*_2,2′_ = ^2^*J*_7,7′_ = 15.9 Hz), 2.19 (d, 2H, 2′- and 7′-H), 2.48 (s, 3H, thienyl-CH_3_), 2.57 (s, 3H, *p*-tolyl CH_3_), 5.51 (s, 1H, 9-H), 6.64 (d, 1H, thienyl 4″-H, ^3^*J*_4″, 5″_ = 5.1 Hz), 6.89 (d, 1H, thienyl 5″-H), 7.08–7.32 (m, 4H, aryl); ^13^C-NMR (CDCl_3_) δ: 14.30 (thienyl-CH_3_), 21.53 (*p*-tolyl CH_3_), 26.43 (C9), 26.89 (2 × CH_3_), 30.05, 30.11, 32.58, 41.91 (C4 and C5), 50.44 (C2 and C7), 115.38–144.59 (C1a, C8a, hetaryl and aryl), 149.89 (C4a and C5a), 195.91 (C1 and C8); IR (KBr) νmax/cm^−1^: 3052 (aromatic, =CH), 2951 and 2867 (CH_3_), 1640 (C=O), 1573 (C=C), 1512 and 1424 (aromatic, C=C); EIMS *m*/*z*: 460 [M + 1, 6%], 459 [M+, 15%], 444 [M+ –(CH_3_), 12%], 430 [M+ –(CO), 2%], 362 [M+ –(thienyl-CH_3_), 100%].

### 2.5. Isolation of Tetrahydroacridinone By-Products ***6a***–***c*** (Scheme 4)

3,3-Dimethyl-7-methoxy-1,2,3,4-tetrahydroacridine-1-one (6a, C_16_H_17_NO_2_)

This compound was isolated as a by-product in 14% yield as colorless crystals, mp 205 °C; ^1^H-NMR (CDCl_3_) δ: 1.12 (s, 6H, 2 × CH_3_), 2.68 (s, 2H, 4-H), 3.52 (s, 2H, 2-H), 3.98 (s, 3H, OCH_3_), 7.39 (d, 1H, 8-H, 4*J*6,8 = 2.7 Hz), 7.68 (dd, 1H, 6-H, ^3^*J*_5,6_ = 9.4 Hz, ^4^*J*_6,8_ = 2.7 Hz), 8.62 (d, 1H, 5-H, ^3^*J*_5,6_ = 9.4 Hz), 9.19 (s, 1H, 9-H); ^13^C-NMR (CDCl_3_) δ: 28.29 (2 × CH_3_), 33.18 (C3), 41.26 (C4), 51.84 (C2), 56.48 (OCH_3_), 107.26 (C8), 123.67 (C6), 130.58 (C5), 136.71 (C8-**C**-C9), 140.40 (C1-**C**-C9), 142.31 (C9), 142.66 (C5-**C**-N), 156.38 (C7), 160.48 (C4-C-N), 193.70 (C1); IR (KBr) νmax/cm^−1^: 3044 (aromatic, =CH), 2946 and 2869 (CH_3_), 1702 (C=O), 1629 (C=N), 1614, 1587 and 1496 (aromatic, C=C); EIMS *m*/*z*: 256 [M + 1, 19%], 255 [M+, 100%], 240 [M+ –(CH_3_), 12%], 227 [M+ –(CO), 12%].

3,3-Dimethyl-7-ethoxy-1,2,3,4-tetrahydroacridine-1-one (6b, C_17_H_19_NO_2_)

This compound was isolated as a by-product in 15% yield as colorless crystals, mp 210 °C; ^1^H-NMR (CDCl_3_) δ: 1.10 (s, 6H, 2 × CH_3_), 1.43 (t, 3H, OCH_2_CH_3_), 2.58 (s, 2H, 4-H), 3.50 (s, 2H, 2-H), 4.05 (q, 2H, OCH_2_CH_3_), 7.36 (d, 1H, 8-H, ^4^*J*_6,8_ = 2.7 Hz), 7.66 (dd, 1H, 6-H, ^3^*J*_5,6_ = 9.4 Hz, ^4^*J*_6,8_ = 2.7 Hz), 8.60 (d, 1H, 5-H, ^3^*J*_5,6_ = 9.4 Hz), 9.21 (s, 1H, 9-H); ^13^C-NMR (CDCl_3_) δ: 15.03 (OCH_2_**C**H_3_), 28.20 (2 × CH_3_), 33.20 (C3), 41.23 (C4), 51.81 (C2), 64.00 (O**C**H_2_CH_3_), 107.22 (C8), 123.75 (C6), 130.51 (C5), 136.70 (C8-**C**-C9), 141.40 (C1-**C**-C9), 142.35 (C9), 142.56 (C5-**C**-N), 156.40 (C7), 160.52 (C4-**C**-N), 192.75 (C1); IR (KBr) νmax/cm^−1^: 3042 (aromatic, =CH), 2946 and 2866 (CH_3_), 1702 (C=O), 1630 (C=N), 1611, 1577 and 1495 (aromatic, C=C); EIMS *m*/*z*: 270 [M + 1, 18%], 269 [M+, 100%], 254 [M+ –(CH_3_), 11%], 241 [M+ –(CO), 13%].

3,3-Dimethyl-7-methyl-1,2,3,4-tetrahydroacridine-1-one (6c, C_16_H_17_NO)

This compound was isolated as a by-product in 13% yield as colorless crystals, mp 192 °C; ^1^H-NMR (CDCl_3_) δ: 1.08 (s, 6H, 2 × CH_3_), 2.43 (s, 2H, 4-H), 2.57 (s, 3H, *7*-CH_3_), 3.45 (s, 2H, 2-H), 7.35 (d, 1H, 8-H, ^4^*J*_6,8_ = 2.7 Hz), 7.67 (dd, 1H, 6-H, ^3^*J*_5,6_ = 9.4 Hz, ^4^*J*_6,8_ = 2.7 Hz), 8.63 (d, 1H, 5-H, ^3^*J*_5,6_ = 9.4 Hz), 9.17 (s, 1H, 9-H); ^13^C-NMR (CDCl_3_) δ: 21.53 (*7*-CH_3_), 28.25 (2 × CH_3_), 33.17 (C3), 41.22 (C4), 51.85 (C2), 107.27 (C8), 123.65 (C6), 130.62 (C5), 136.77 (C8-**C**-C9), 140.10 (C1-**C**-C9), 142.41 (C9), 142.63 (C5-**C**-N), 156.37 (C7), 160.47 (C4-**C**-N), 193.50 (C1); IR (KBr) νmax/cm^−1^: 3042 (aromatic, =CH), 2945 and 2868 (CH_3_), 1702 (C=O), 1629 (C=N), 1612, 1585 and 1493 (aromatic, C=C); EIMS *m*/*z*: 240 [M + 1, 18%], 239 [M+, 100%], 224 [M+ –(CH_3_), 12%], 211 [M+ –(CO), 12%]. The δ 2.57 ppm resonance is assigned to 7-CH_3_, the arylamine-derived ring methyl; an intact p-tolyl or thienyl-methyl substituent is not present in isolated **6c**.

**Scheme 4:**
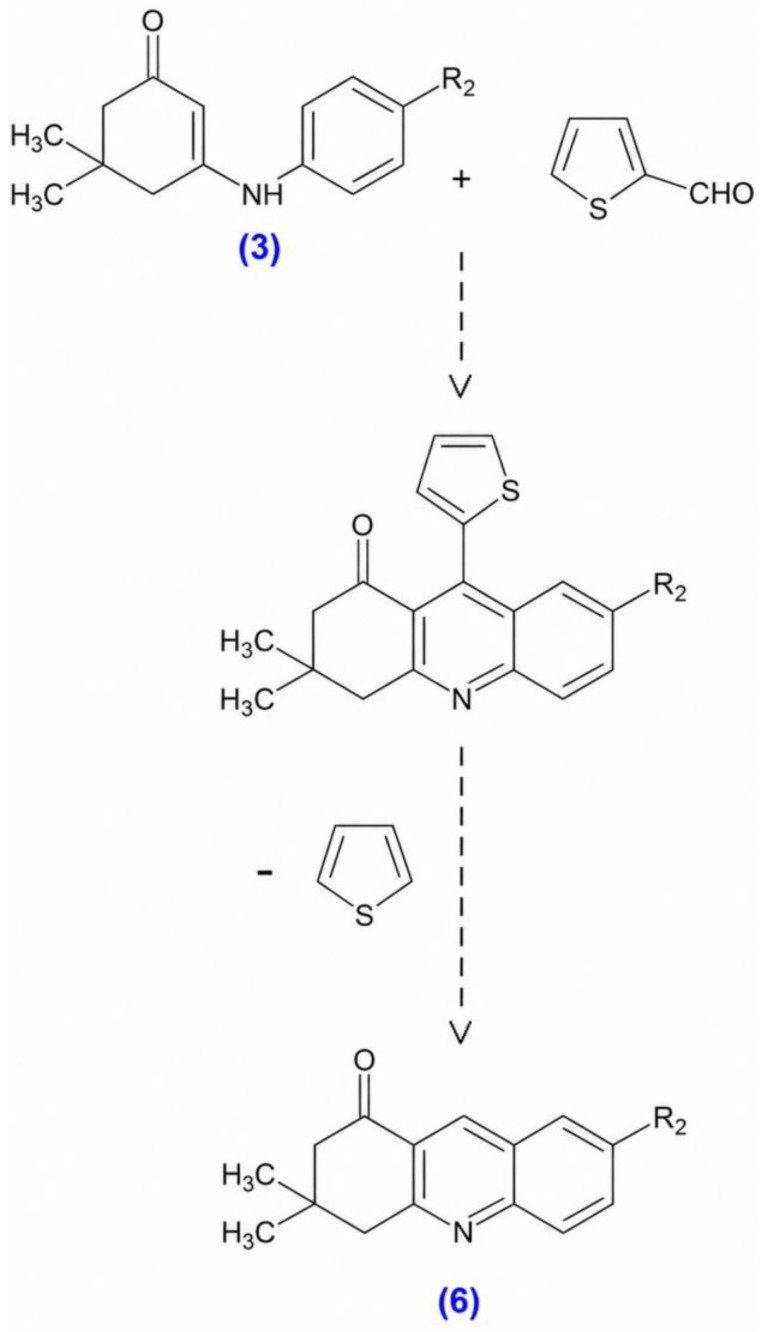
One hypothetical and nonexclusive pathway for the by-product manifold: enaminones **3a**–**c** and heteroaryl aldehydes **4a**,**b** under the multicomponent conditions; isolated tetrahydroacridinone products are designated **6a**–**c**.

## 3. Results and Discussion

Procedures II and III used the same Brønsted-acid catalyst concentration (0.016 M), solvent, temperature, and heating period, but differed in substrate concentration and catalyst mol% relative to the limiting substrates. The comparison should, therefore, be interpreted as an operational comparison of two archived preparative sequences, not as an isomolar kinetic experiment or proof that sequence alone caused the yield difference. Within this limitation, the product-forming second stage showed a consistent 28–33 percentage-point advantage across the six matched substrate combinations, whereas the calculated overall two-step yields remained higher than the corresponding one-pot yields.

### 3.1. Effect of Reaction Sequence on Isolated Yield

The isolated yields of the product-forming second stage were 71–75%, corresponding to calculated overall two-step yields of 58.2–68.6% after accounting for the isolated enaminone-preparation yields (Table 1). These overall yields remained higher than those of the one-pot procedure (40–47%), although the sequential protocol required an additional reaction, isolation, catalyst charge, and solvent-intensive operation. Because the historical procedures were not identical in concentration and catalyst mol% relative to the limiting substrates, the result must not be interpreted as a kinetic rate comparison or as unambiguous proof that the component sequence was the sole causal variable. This establishes the practical superiority of the complete archived sequential protocol within the investigated *p*-toluenesulfonic acid/glacial acetic acid conditions. No comparative experiments in ethanol, acetonitrile, or another solvent were performed; solvent-independent superiority is, therefore, not claimed.

Within procedure II, the 2-thienyl series **5a**–**c** averaged 74.3%, whereas the 3-methyl-2-thienyl series **5d**–**f** averaged 72.0%. This small decrease may reflect additional steric demand adjacent to the aldehyde-derived carbon, but the limited series does not support a strong substituent-effect conclusion. Similarly, variation of the para N-aryl substituent from methoxy to ethoxy or methyl produced only small and nonmonotonic yield changes. The multicomponent series also showed no simple electronic trend, consistent with several competing elementary steps rather than a single rate- or selectivity-determining event.

### 3.2. Structural Evidence for Target Compounds ***5a***–***f***

**Figure 1:**
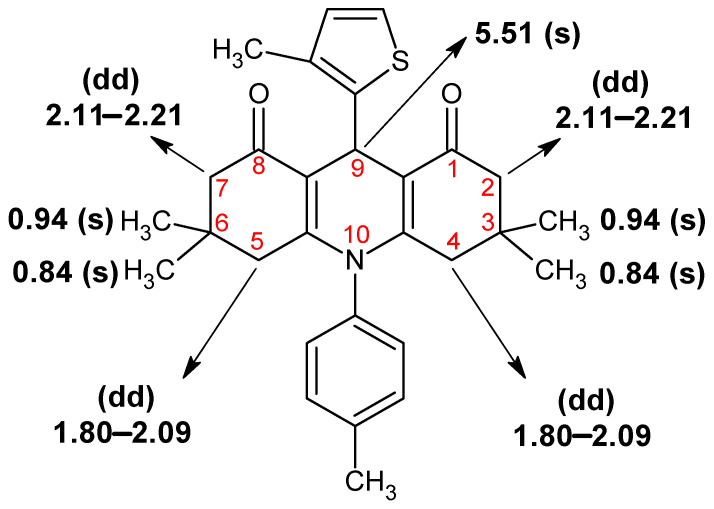
^1^H NMR chemical-shift assignments (δ, ppm) for representative compound **5f**; red numerals indicate carbon-atom numbering.

**Figure 2:**
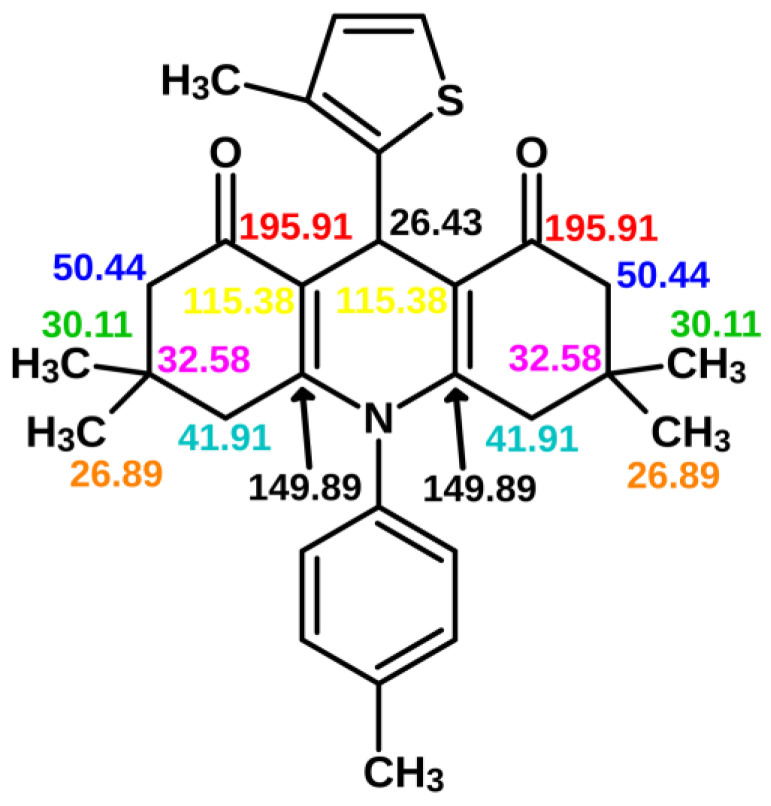
^13^C NMR chemical-shift assignments (δ, ppm) for representative compound **5f**; identical colors denote symmetry-equivalent carbon environments and are used solely as visual guides.

The NMR data are consistent with the formation of a symmetric decahydroacridine-1,8-dione core (Figure 1 and Figure 2). Compounds **5a**–**f** show two sets of gem-dimethyl resonances and four pairs of ring-methylene protons. The latter appear as coupled AB-type signals because the two protons of each methylene group are diastereotopic in the rigid fused framework. The aldehyde-derived C-9 proton gives a diagnostic singlet at 5.51–5.66 ppm. In representative compound **5f**, the assignments displayed in Figure 1 and Figure 2 illustrate the bilateral equivalence of C-1/C-8, C-2/C-7, C-3/C-6 and C-4/C-5 and support the proposed substitution pattern.

**Figure 3:**
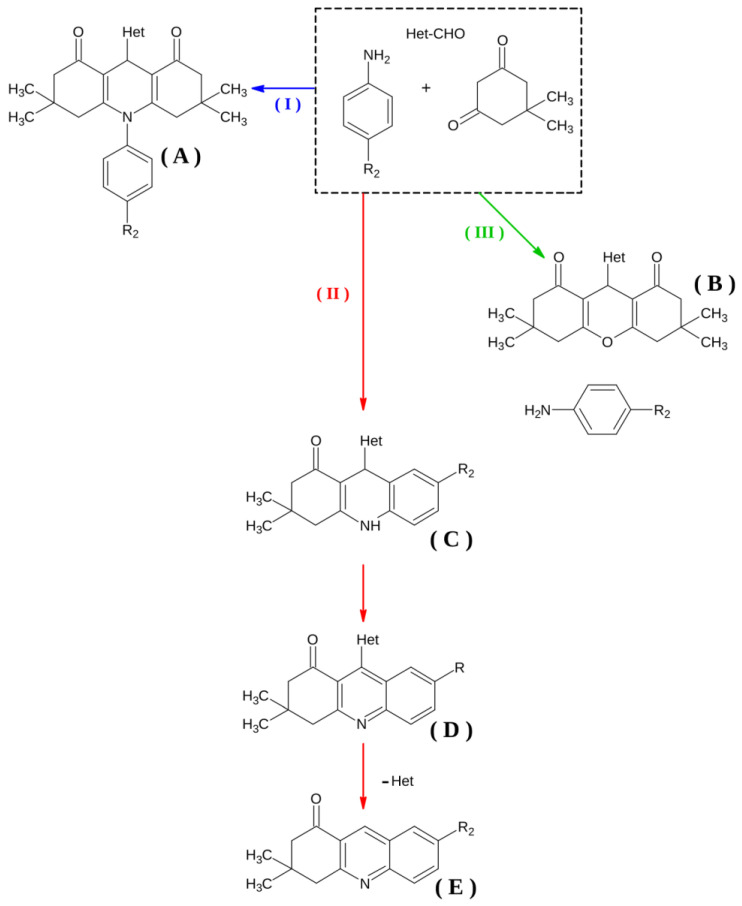
Proposed, nonexclusive connectivity network for the aldehyde–dimedone–amine system. (**A**) denotes the isolated target class **5a**–**f** and (**E**) denotes the isolated by-product class **6a**–**c**. B is depicted as an independent, tentative xanthene-type competing sink, whereas (**C**–**E**) constitute a separate mono-dimedone nitrogen-heterocycle branch. (**B**–**D**) were not isolated as pure compounds; tentative trace nominal-mass EI-MS evidence is available only for (**B**,**D**) in the Supporting Information.

**Figure 4:**
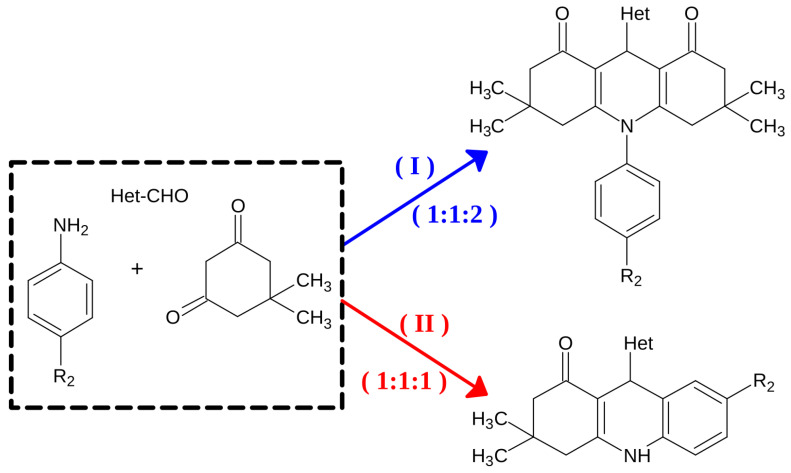
Proposed stoichiometric control of product formation. Pathway I uses a 1:1:2 aldehyde/amine/dimedone ratio to furnish the symmetric decahydroacridine-1,8-dione framework, whereas the competing 1:1:1 manifold can lead to mono-dimedone acridinone-type products.

The proposed connectivity network and stoichiometric control are shown in Figure 3 and Figure 4, respectively.

The carbonyl groups of **5a**–**f** absorb at 1634–1645 cm^−1^ in the IR spectra and resonate near 195.8 ppm in the ^13^C NMR spectra. EI-MS provides an additional distinction between the two heteroaryl series. For **5a**–**c**, the molecular ion is the base peak and loss of a thienyl fragment gives an abundant [M-83]^+^ ion. For **5d**–**f**, the molecular ions are weaker, and loss of the 3-methylthienyl fragment produces the base peak [M-97]^+^. The systematic 14 mass-unit shift between the two fragment series independently tracks the methyl substituent on the thiophene ring and supports retention of the heteroaryl group in the target products.

### 3.3. Formation and Identification of By-Products ***6a***–***c***

The isolated by-products **6a**–**c** differ from **5a**–**f** in both carbon count and degree of unsaturation. Their IR carbonyl bands occur at 1702 cm^−1^, substantially higher than those of the conjugated 1,8-dione products, and each spectrum contains a C=N-associated band at 1629–1630 cm^−1^. In the ^1^H NMR spectra, the C-9 methine singlet at approximately 5.5–5.7 ppm is absent and a strongly deshielded singlet appears at 9.17–9.21 ppm. This singlet is assigned to H-9 from the one-dimensional aromatic coupling pattern: H-5 is an ortho-coupled doublet (*J* approximately 9.4 Hz), H-6 is a doublet of doublets (*J* approximately 9.4 and 2.7 Hz), and H-8 is a meta–coupled doublet (*J* approximately 2.7 Hz), leaving H-9 as the isolated aromatic proton. The assignment is strongly structure-consistent but is not described as definitive because no HSQC or HMBC data are available. The ^13^C NMR spectra contain a single carbonyl resonance at 192.75–193.70 ppm, and the molecular ions at *m*/*z* 255, 269, and 239 support the assigned mono-dimedone tetrahydroacridinone structures.

By-products 6a–c were obtained in similar yields (13–15%, Table 2), indicating that methoxy, ethoxy, and methyl substitution on the arylamine-derived ring has only a modest effect on this branch under the investigated conditions. The isolated products contain no thiophene-derived fragment. However, the mother liquors were not subjected to exhaustive chromatographic fractionation, quantitative aldehyde recovery, or a complete carbon-balance analysis; the fate of the heteroaryl aldehyde, therefore, cannot be assigned uniquely. Plausible destinations include unreacted aldehyde, aldehyde–dimedone Knoevenagel adducts, bis-dimedone/xanthene-type products such as proposed B, non-isolated heteroaryl-containing nitrogen heterocycles such as proposed D, and unresolved degradation or oligomeric material. The formation of **6a**–**c** is compatible either with exclusion of the aldehyde from that branch or with loss of a heteroaryl-containing unit after initial incorporation, and the available data do not discriminate between these alternatives.

### 3.4. Plausible Mechanism and Stoichiometric Control

For the productive sequential route, *p*-toluenesulfonic acid is proposed to activate the heteroaryl aldehyde toward nucleophilic attack by the β-carbon of preformed enaminone 3. Proton transfer and dehydration generate a conjugated electrophile, which is trapped by the enol form of dimedone through Michael addition. Subsequent intramolecular C-N bond formation, dehydration and tautomerization furnish the 1,8-dioxo-decahydroacridine framework. This general order of Knoevenagel-type activation, Michael addition and annulation is consistent with established acridinedione syntheses [14,15,16,17,18,20], while preformation of the enaminone reduces the number of independently competing species present at the outset.

In procedure III, aldehyde, arylamine, and dimedone are exposed simultaneously to the acidic medium. Aldehyde–dimedone condensation, amine–dimedone enaminone formation, and 1:1:1 nitrogen-heterocycle formation may, therefore, occur in parallel before complete formation of enaminone 3. Addition of a second dimedone to an alkylidene-dimedone intermediate may lead toward the desired carbon skeleton, whereas an independent oxygen-centered branch may furnish bis-dimedone/xanthene-type material. A separate mono-dimedone nitrogen-heterocycle manifold may undergo dehydration, dehydrogenation, and aromatization to give acridinone-type products. The relative rates of these competing events were not measured, and the one-pot route is not claimed to proceed through a single obligatory enaminone intermediate.

In the revised network shown in Figure 3, A denotes the isolated decahydroacridine-1,8-dione products **5a**–**f** and E denotes the isolated tetrahydroacridinones **6a**–**c**. B is an independent, tentative xanthene-type competing sink formed from aldehyde and two dimedone units; no direct B-to-C transformation is proposed. C and D belong to a separate mono-dimedone nitrogen-heterocycle branch that may converge on E. The scheme is a nonexclusive connectivity model originating from a common reagent pool and does not require every branch to pass through isolated enaminone 3. B–D were not independently isolated, C was not directly detected, and more than one pathway may converge on E. 

### 3.5. Scope and Limitations of the Mechanistic Assignment

The principal strength of the study is the paired preparative comparison of two assembly sequences together with characterization of the major isolable by-product class. Trace nominal-mass EI-MS features consistent with proposed species B and D were observed in archived reaction-mixture data (Figures S29 and S30), but neither species was isolated as a pure compound, no crude-reaction ^1^H NMR spectrum or diagnostic xanthene-type methine resonance could be recovered from the surviving post-doctoral and laboratory archives, and constitutional isomers or co-eluting species cannot be excluded. Species C was not directly detected. The reactions were followed qualitatively by TLC, but no defined 1, 2, and 4 h TLC records, densitometric data, conversion measurements, aliquot-based kinetic profiles, complete mass balance, comparative solvent controls, time-resolved NMR, independent intermediate synthesis, isotope labeling, or kinetic experiments are available. Consequently, the historical dataset cannot quantitatively distinguish incomplete conversion, degradation, and diversion into competing pathways. The network is, therefore, an evidence-based, nonexclusive interpretation inferred from stoichiometry, isolated product structures, tentative trace mass-spectral evidence, and established dimedone/enaminone reactivity.

## 4. Conclusions

A sequential preformed-enaminone route to six 9-thienyl-10-aryl decahydroacridine-1,8-diones **5a**–**f** was evaluated against the corresponding one-pot multicomponent process. Under the investigated *p*-toluenesulfonic acid/glacial acetic acid protocols, the isolated yields of the product-forming second stage were 71–75%, corresponding to calculated overall two-step yields of 58.2–68.6%, compared with 40–47% for the multicomponent route. These overall yields remained higher, although the sequential protocol required an additional reaction, isolation, catalyst charge, and solvent-intensive operation. The archived protocols differed in substrate concentration and effective catalyst loading relative to the limiting substrates; therefore, the observed yield differences cannot be attributed unambiguously to component sequence alone. Available IR, ^1^H NMR, ^13^C NMR, and nominal-mass EI-MS data support the assigned product classes and distinguish **5a**–**f** from tetrahydroacridinone by-products 6a–c. The proposed network rationalizes the observed product distribution but is not a demonstrated linear mechanism. Verification of individual intermediates and solvent-independent generality will require new controlled concentration/catalyst studies, comparative solvent experiments, in situ monitoring, labeling, or independent synthesis.

## Figures and Tables

**Table 1:** Isolated yields of target compounds **5a**–**f** for the product-forming stage of the sequential enaminone route (procedure II), calculated overall two-step yields for procedures I and II, and isolated yields from the one-pot multicomponent route (procedure III).

Compound	R_1_	R_2_	Yield (Procedure II: Product-Forming Stage)	Calculated Overall Two-Step Yield ^a^	Yield (Procedure III: One-Pot Multicomponent)
**5a**	H	OMe	73%	68.6%	45%
**5b**	H	OEt	75%	63.8%	43%
**5c**	H	Me	75%	61.5%	47%
**5d**	Me	OMe	73%	68.6%	40%
**5e**	Me	OEt	72%	61.2%	42%
**5f**	Me	Me	71%	58.2%	42%

^a^ Calculated overall two-step yield = (isolated enaminone-preparation yield from procedure I × isolated product-forming-stage yield from procedure II)/100.

**Table 2:** Isolated tetrahydroacridinone by-products **6a**–**c** under the investigated conditions. A dash indicates that the corresponding product was not isolated.

Compound	R_1_	R_2_	Yield (Procedure II: Enaminone)	Yield (Procedure III: Multicomponent)
**6a**	-	OMe	-	14%
**6b**	-	OEt	-	15%
**6c**	-	Me	-	13%

## Data Availability

All surviving data supporting this study are included in the article and Supporting Information. The work was carried out during the author’s post-doctoral research and subsequent continuation studies approximately two decades ago. A systematic search of the surviving post-doctoral and laboratory archives was undertaken. Every original instrument printout that could be recovered is reproduced in the Supporting Information. The original printouts, raw electronic files, and residual samples for **5b**, **5d**, **5e**, **6b**, and **6c** could not be recovered from those archives. The reported numerical values were transcribed contemporaneously into the original laboratory records and were not reconstructed for the present manuscript. No unavailable spectrum or integral trace has been simulated or presented as original experimental data.

## Supplementary Materials

The following supporting information can be downloaded at: https://www.mdpi.com/article/10.3390/molecules31152680/s1.
