# Supplementary material for: Sequence-Controlled Synthesis of Heteroaryl-Substituted Decahydroacridine-1,8-diones: Comparative Evaluation of Preformed Enaminone and Multicomponent Routes
Source: Molecules. 2026 Jul 31;31(15):2680. doi: 10.3390/molecules31152680 (PMC13467679; doi:10.3390/molecules31152680)
Supplement: Supplementary file 1 [file molecules-31-02680-s001.zip › molecules-4465362-supplementary.pdf]

SUPPORTING INFORMATION

Sequence-Controlled Synthesis of Heteroaryl-Substituted Decahydroacridine-1,8-diones: Comparative Evaluation of Preformed Enaminone and Multicomponent Routes

Gökhan Özokan

Department of Chemistry, Faculty of Science, Yildiz Technical University, Davutpasa Campus, Istanbul 34220, Türkiye

ORCID: 0000-0003-1140-1996

Archived IR, ^{1}H NMR, ^{13}C NMR and EI-MS Data

Historical data and integrity statement

The synthetic work described in this study was carried out during the author's post-doctoral research and in the subsequent continuation of that research approximately two decades ago. During preparation of the present manuscript, a systematic search of the surviving post-doctoral and laboratory archives was undertaken. This file reproduces every original instrument printout that could be recovered for compounds 3a-c, 5a, 5c, 5f, and 6a. For compounds 5b, 5d, 5e, 6b, and 6c, the original printouts, raw electronic data, and residual samples could not be recovered from the surviving post-doctoral and laboratory archives; only numerical data transcribed contemporaneously into the original laboratory records are reported. No unavailable spectrum has been reconstructed, simulated, or presented as an original experimental spectrum. Trace nominal-mass EI-MS data for proposed species B and D are included only as tentative mechanistic support; those species were not isolated as pure compounds.

Contents

Compound/data set	Material included	Data status
3a, 3b, 3c	IR; ^{1}H NMR; ^{13}C NMR; EI-MS	Original archived printouts
5a, 5c, 5f	IR; ^{1}H NMR; ^{13}C NMR; EI-MS	Original archived printouts
6a	IR; ^{1}H NMR; ^{13}C NMR; EI-MS	Original archived printouts
5b, 5d, 5e	Reported numerical IR; ^{1}H NMR; ^{13}C NMR; EI-MS values	**Original printouts, raw files, and residual samples could not be recovered from the surviving post-doctoral and laboratory archives**
6b, 6c	Reported numerical IR; ^{1}H NMR; ^{13}C NMR; EI-MS values	**Original printouts, raw files, and residual samples could not be recovered from the surviving post-doctoral and laboratory archives**
Proposed species B and D	Trace EI-MS	**Tentative nominal-mass EI-MS assignments; not isolated and not confirmed by NMR**

Important data-status statement. HRMS, elemental-analysis data, multidimensional NMR data, reliable numerical integral traces, and raw FID files are not available and are not concealed. The surviving original ^{1}H NMR printouts do not contain reliable numerical integral traces, and authentic integrations cannot be regenerated or normalized retrospectively without the raw FID files. Reconstructed integral curves have therefore not been added. Instead, the assigned proton count is reported for every resonance in the numerical ^{1}H NMR descriptions. EI-MS is reported as nominal-mass evidence only.

Clarification of Figure 3 nomenclature. The label A in the manuscript's Figure 3 denotes the isolated product class 5a-f; it is not an additional discrete compound whose spectrum is absent. The surviving original spectra for representative members 5a, 5c, and 5f are reproduced below.

Validation of historical numerical records. The transcribed data were checked by direct comparison with the surviving original printouts for related members of the same compound classes; verification of molecular formulas against nominal molecular ions and characteristic fragment losses; examination of proton counts, multiplicities, coupling relationships, symmetry-equivalent carbon environments, and substituent-dependent 14 Da homologous shifts across the 5a-f series; comparison with the enaminone data in Ref. [21] and related acridine-1,8-dione literature cited in the manuscript; and cross-checking among the contemporaneous laboratory records, manuscript, and Supporting Information. This documentary and internally comparative validation tests chemical plausibility but is not equivalent to modern remeasurement or raw-data reprocessing.

Compound 3a - IR

5,5-Dimethyl-3-[(4-methoxyphenyl)amino]cyclohex-2-en-1-one

$C_{15}H_{19}NO_2$

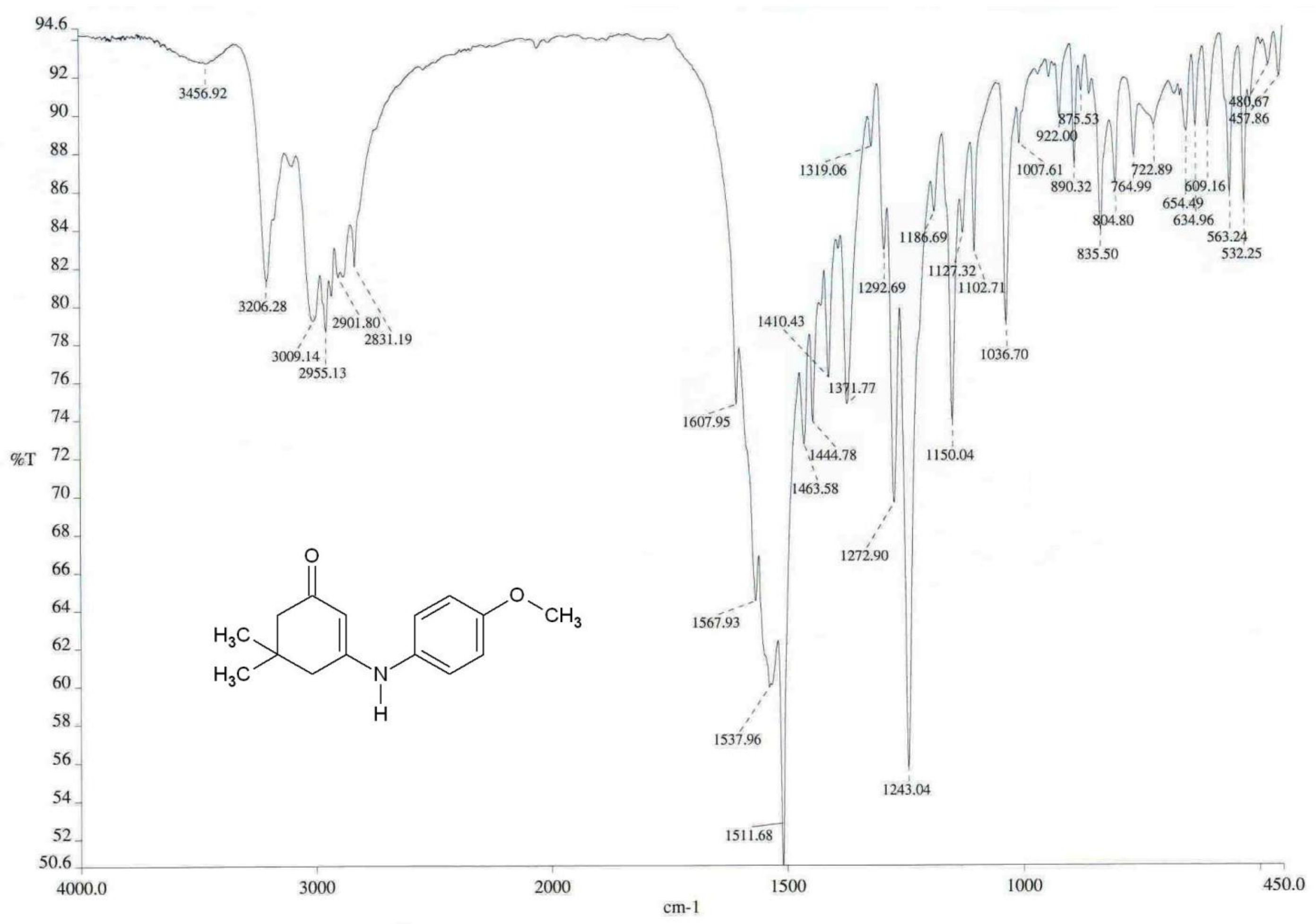

Figure S1. IR spectrum of compound 3a (KBr).

Compound 3a - ¹H NMR

$C_{15}H_{19}NO_2$

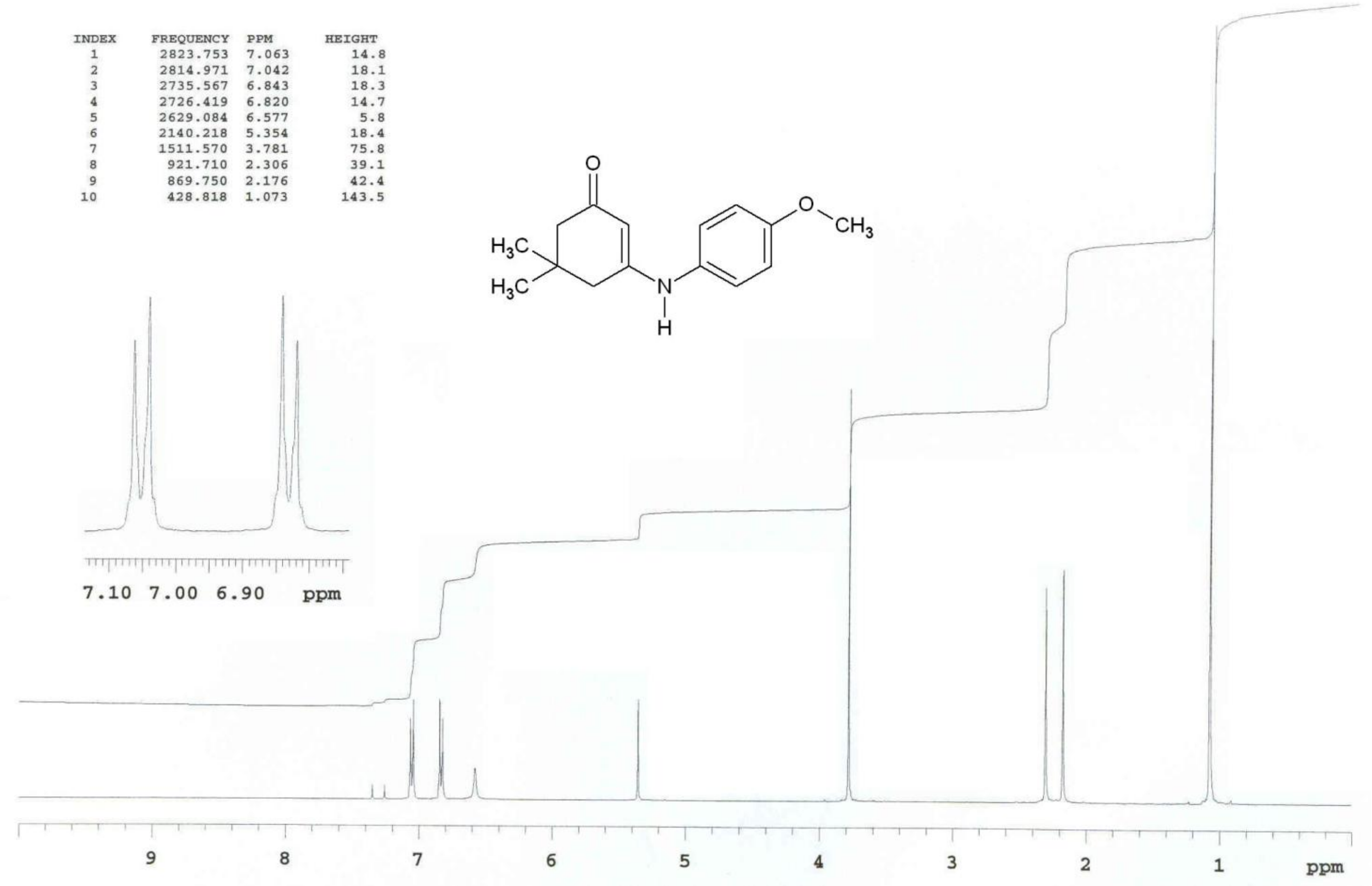

INDEX	FREQUENCY	PPM	HEIGHT
1	2823.753	7.063	14.8
2	2814.971	7.042	18.1
3	2735.567	6.843	18.3
4	2726.419	6.820	14.7
5	2629.084	6.577	5.8
6	2140.218	5.354	18.4
7	1511.570	3.781	75.8
8	921.710	2.306	39.1
9	869.750	2.176	42.4
10	428.818	1.073	143.5

Figure S2. ¹H NMR spectrum of compound 3a ($CDCl_3$).

Compound 3a - ^{13}C NMR

$C_{15}H_{19}NO_2$

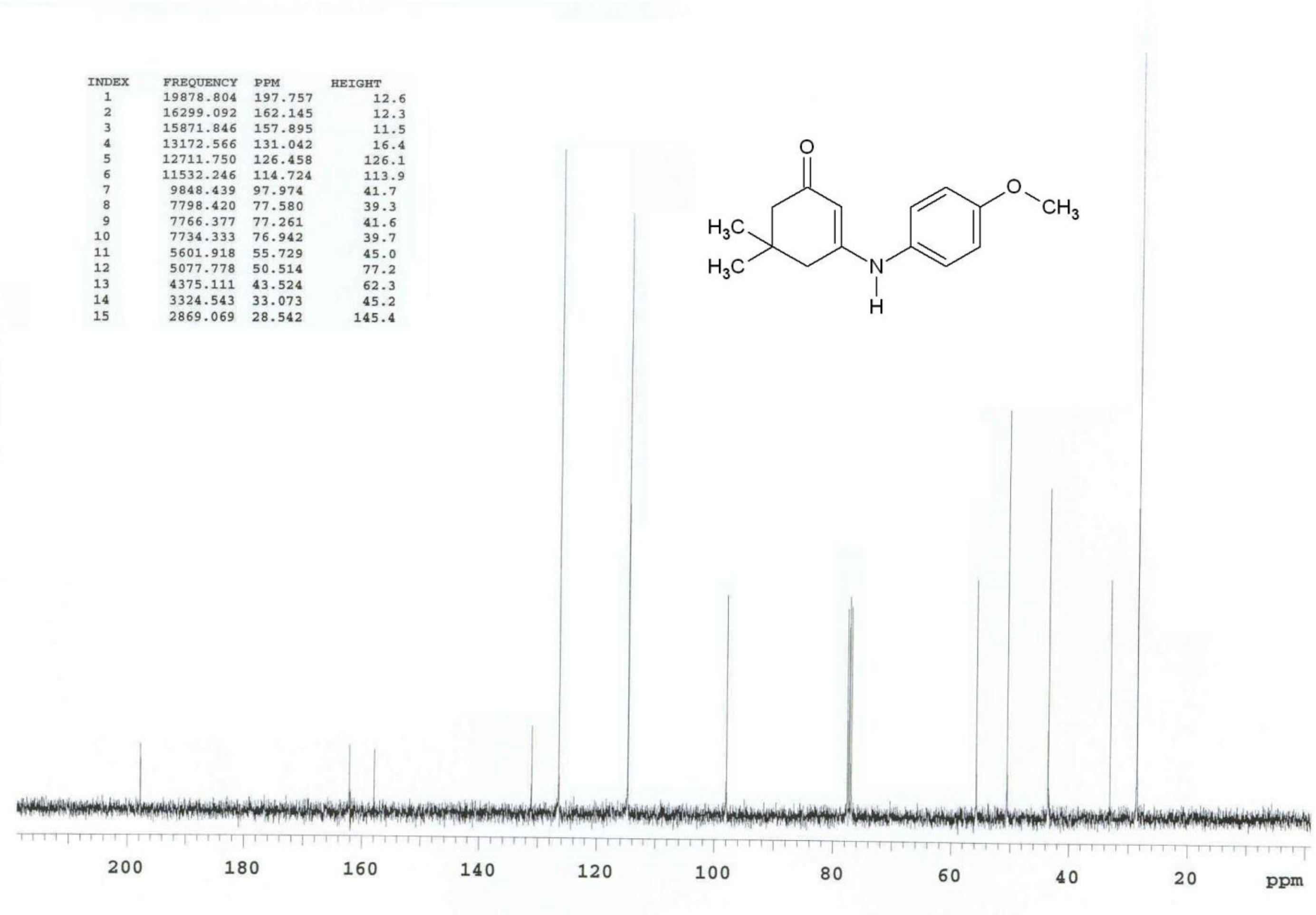

INDEX	FREQUENCY	PPM	HEIGHT
1	19878.804	197.757	12.6
2	16299.092	162.145	12.3
3	15871.846	157.895	11.5
4	13172.566	131.042	16.4
5	12711.750	126.458	126.1
6	11532.246	114.724	113.9
7	9848.439	97.974	41.7
8	7798.420	77.580	39.3
9	7766.377	77.261	41.6
10	7734.333	76.942	39.7
11	5601.918	55.729	45.0
12	5077.778	50.514	77.2
13	4375.111	43.524	62.3
14	3324.543	33.073	45.2
15	2869.069	28.542	145.4

Figure S3. ^{13}C NMR spectrum of compound 3a ($CDCl_3$).

Compound 3a - EI-MS

$C_{15}H_{19}NO_2$

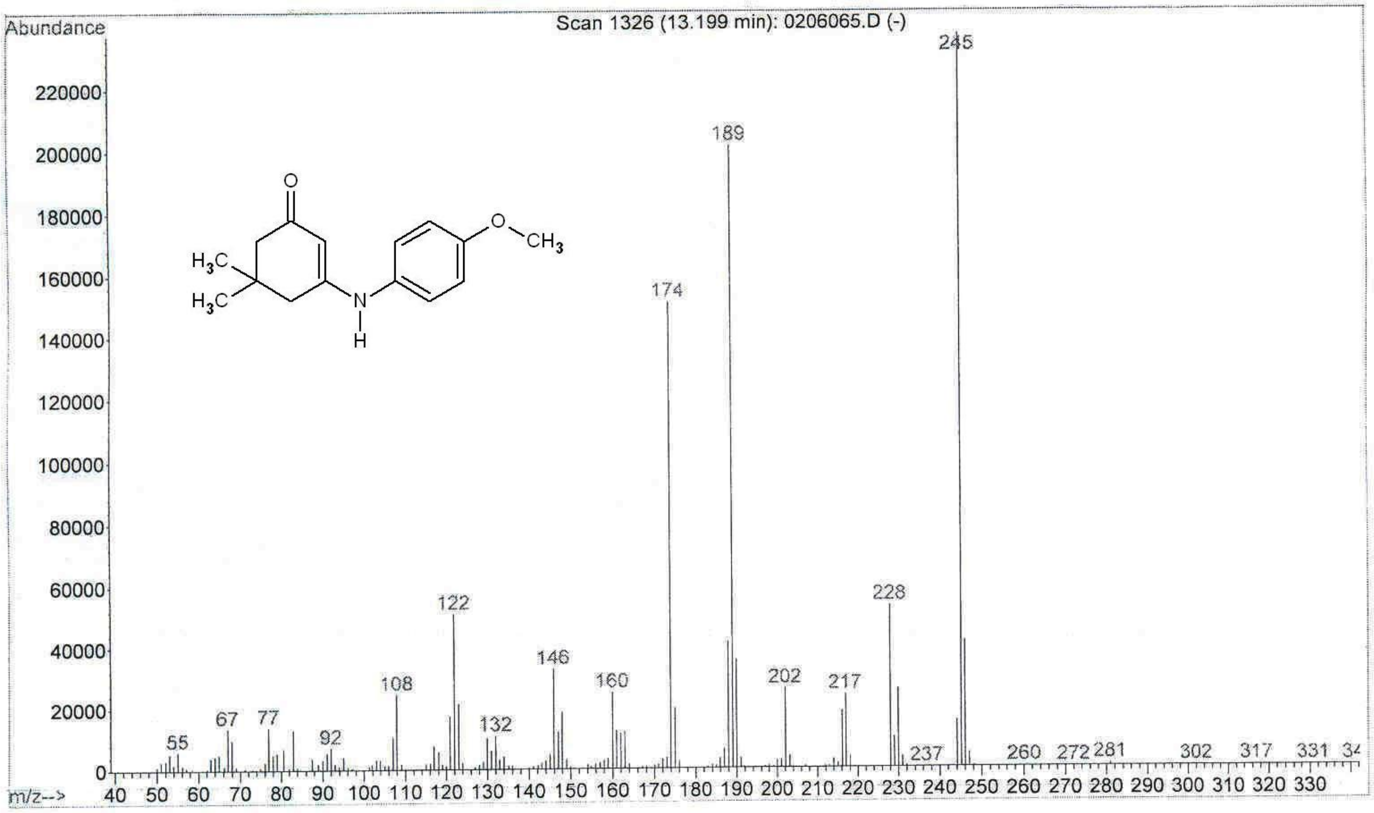

Figure S4. EI mass spectrum of compound 3a.

Compound 3b - IR

5,5-Dimethyl-3-[(4-ethoxyphenyl)amino]cyclohex-2-en-1-one

$C_{16}H_{21}NO_2$

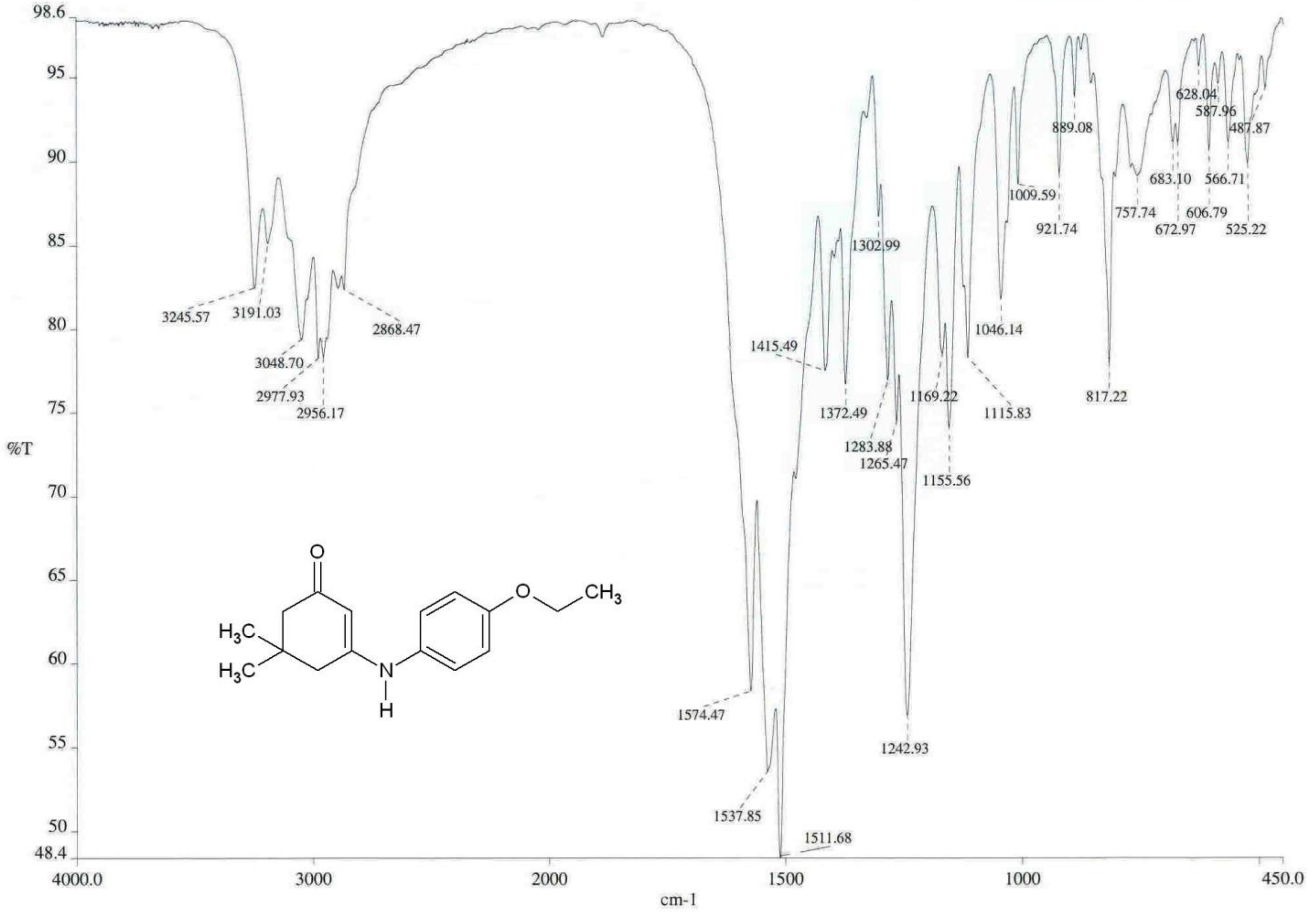

Figure S5. IR spectrum of compound 3b (KBr).

Compound 3b - ¹H NMR

$C_{16}H_{21}NO_2$

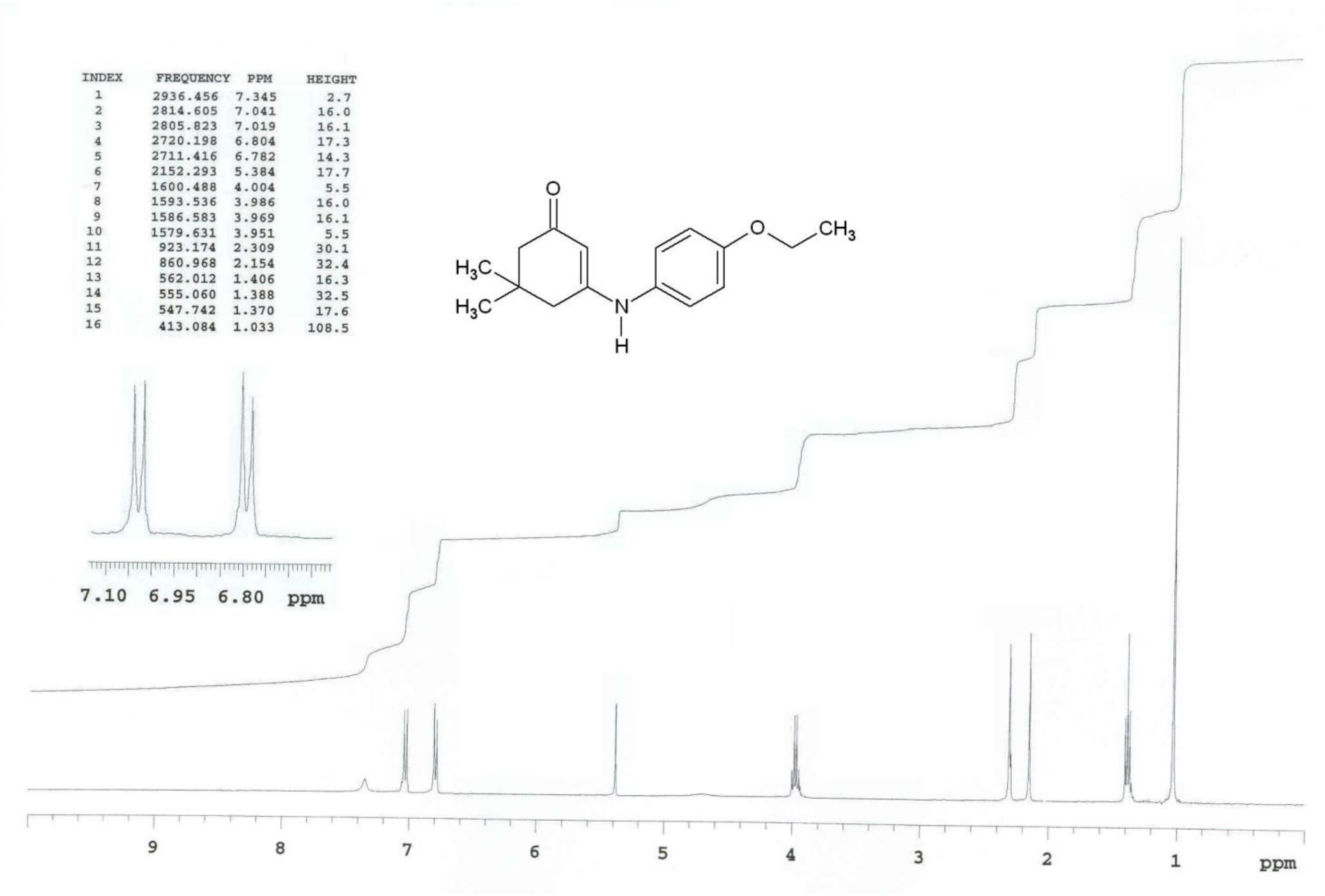

INDEX	FREQUENCY	PPM	HEIGHT
1	2936.456	7.345	2.7
2	2814.605	7.041	16.0
3	2805.823	7.019	16.1
4	2720.198	6.804	17.3
5	2711.416	6.782	14.3
6	2152.293	5.384	17.7
7	1600.488	4.004	5.5
8	1593.536	3.986	16.0
9	1586.583	3.969	16.1
10	1579.631	3.951	5.5
11	923.174	2.309	30.1
12	860.968	2.154	32.4
13	562.012	1.406	16.3
14	555.060	1.388	32.5
15	547.742	1.370	17.6
16	413.084	1.033	108.5

Figure S6. ¹H NMR spectrum of compound 3b ($CDCl_3$).

Compound 3b - ^{13}C NMR

$C_{16}H_{21}NO_2$

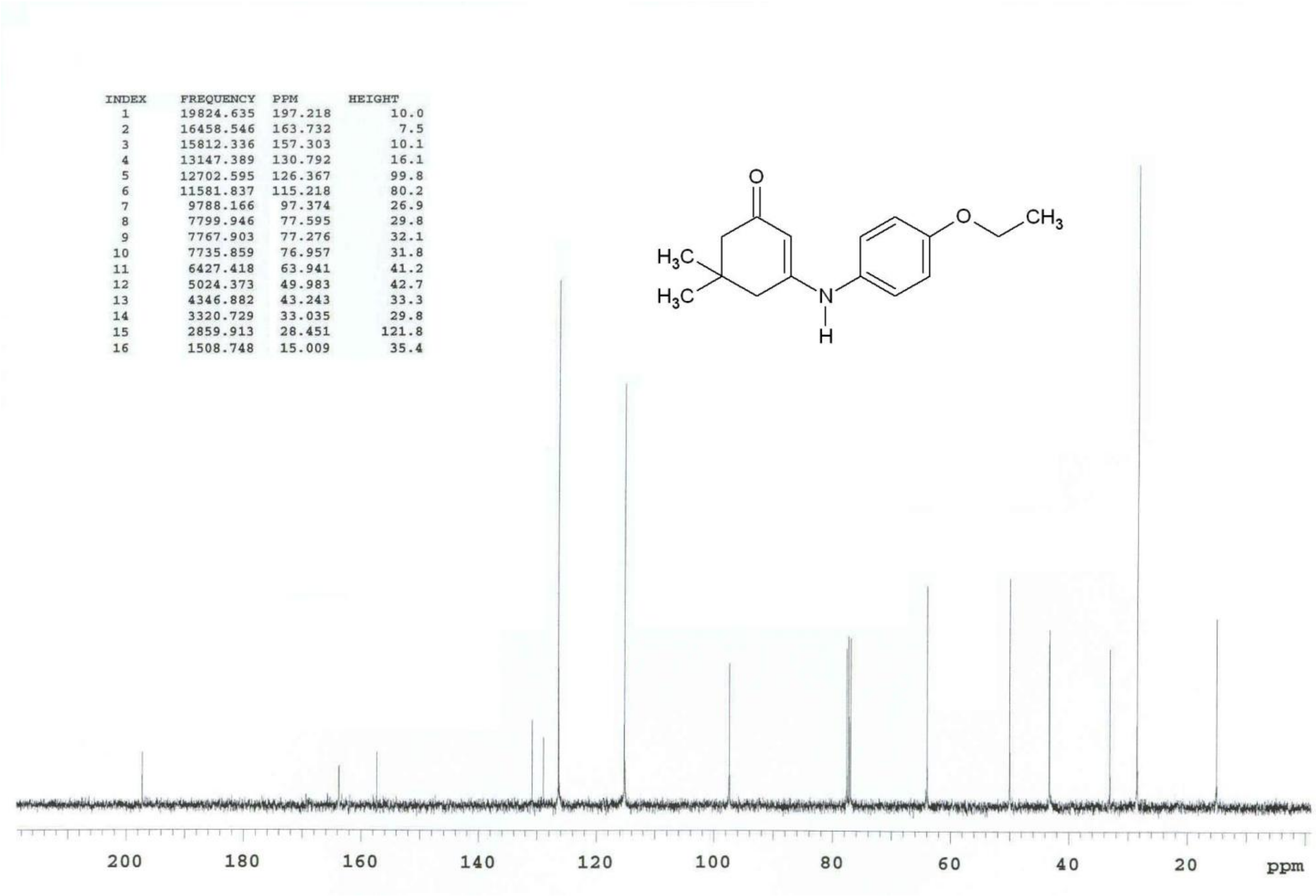

INDEX	FREQUENCY	PPM	HEIGHT
1	19824.635	197.218	10.0
2	16458.546	163.732	7.5
3	15812.336	157.303	10.1
4	13147.389	130.792	16.1
5	12702.595	126.367	99.8
6	11581.837	115.218	80.2
7	9788.166	97.374	26.9
8	7799.946	77.595	29.8
9	7767.903	77.276	32.1
10	7735.859	76.957	31.8
11	6427.418	63.941	41.2
12	5024.373	49.983	42.7
13	4346.882	43.243	33.3
14	3320.729	33.035	29.8
15	2859.913	28.451	121.8
16	1508.748	15.009	35.4

Figure S7. ^{13}C NMR spectrum of compound 3b ($CDCl_3$).

Compound 3b - EI-MS

$C_{16}H_{21}NO_2$

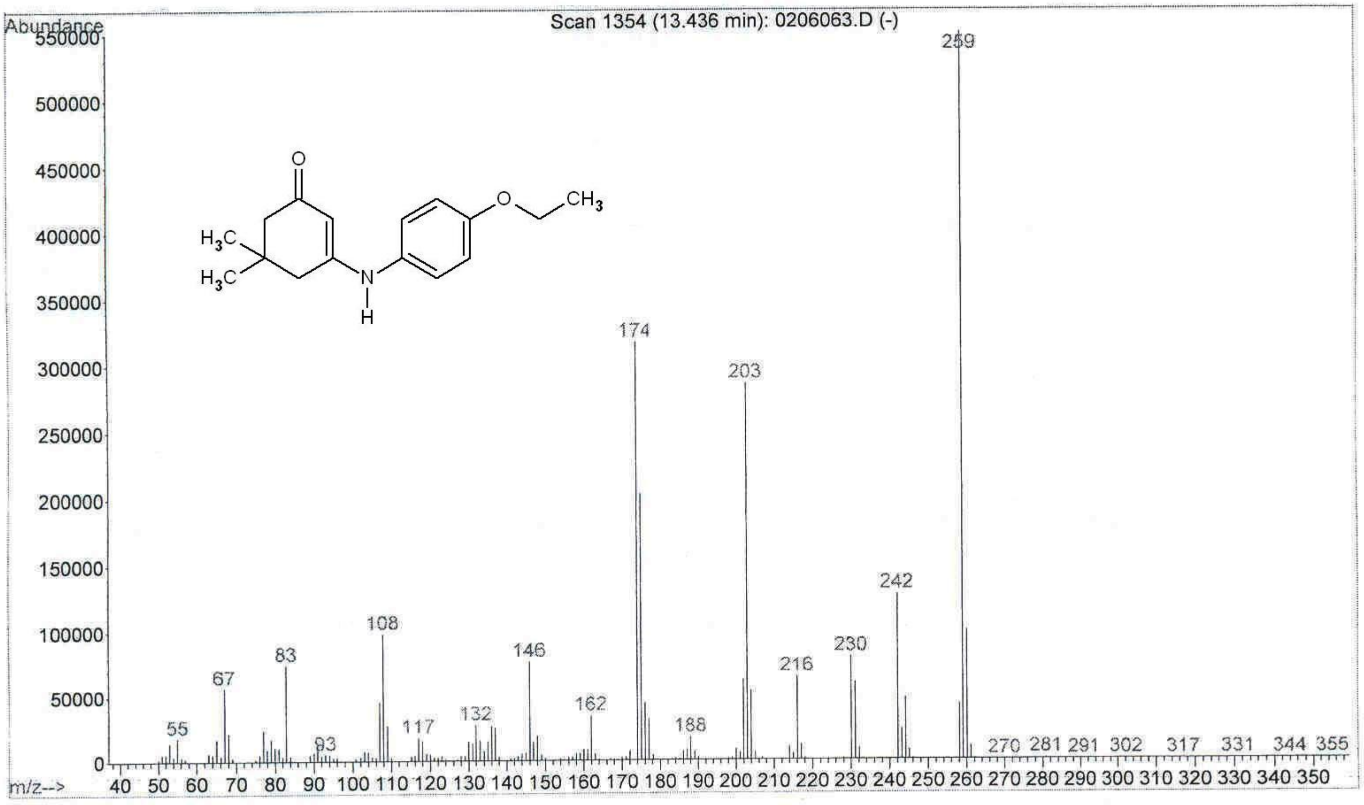

Figure S8. EI mass spectrum of compound 3b.

Compound 3c - IR

5,5-Dimethyl-3-[(4-methylphenyl)amino]cyclohex-2-en-1-one

$C_{15}H_{19}NO$

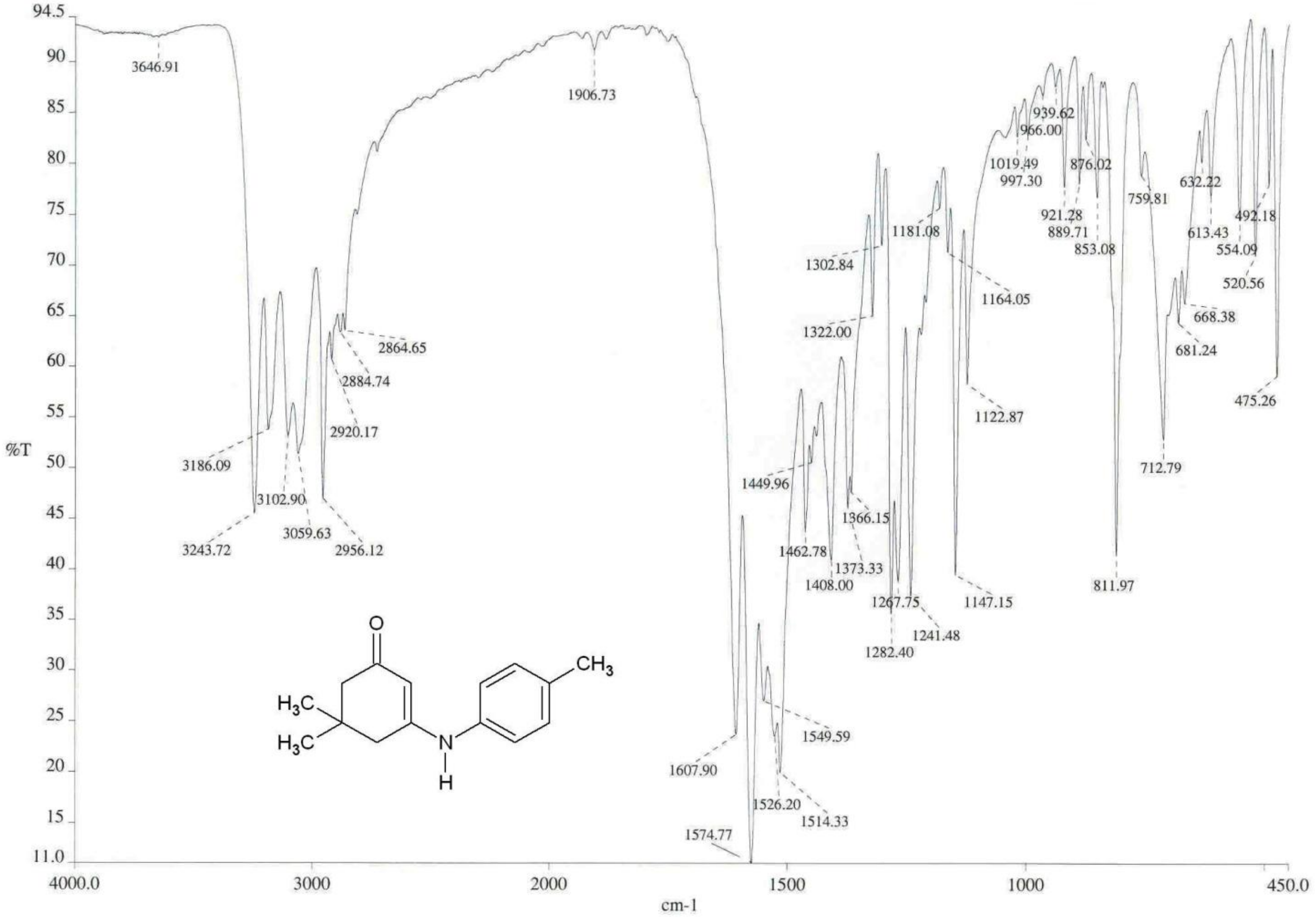

Figure S9. IR spectrum of compound 3c (KBr).

Compound 3c - ¹H NMR

$C_{15}H_{19}NO$

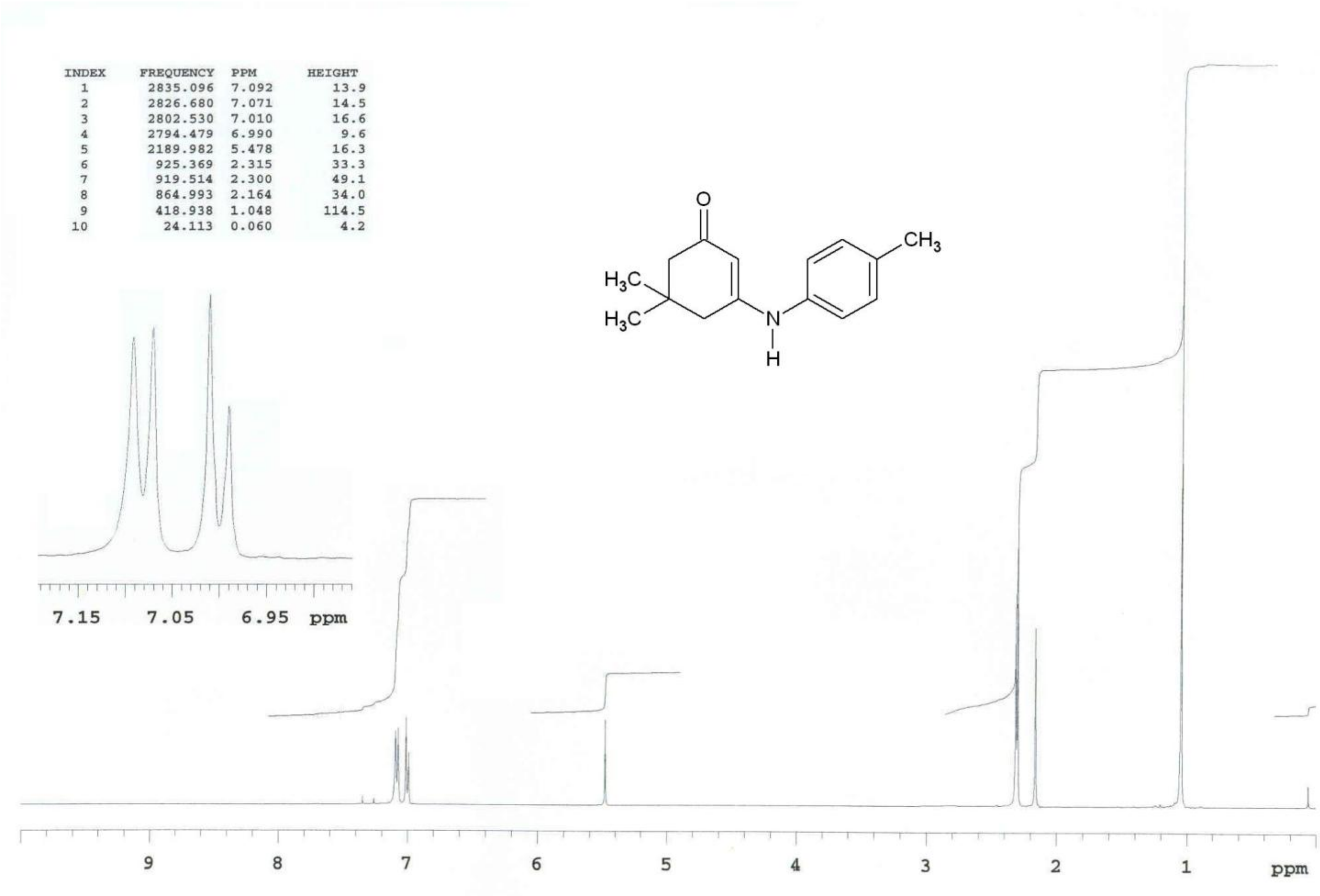

Figure S10. ¹H NMR spectrum of compound 3c (CDCl₃).

Compound 3c - ¹³C NMR

$C_{15}H_{19}NO$

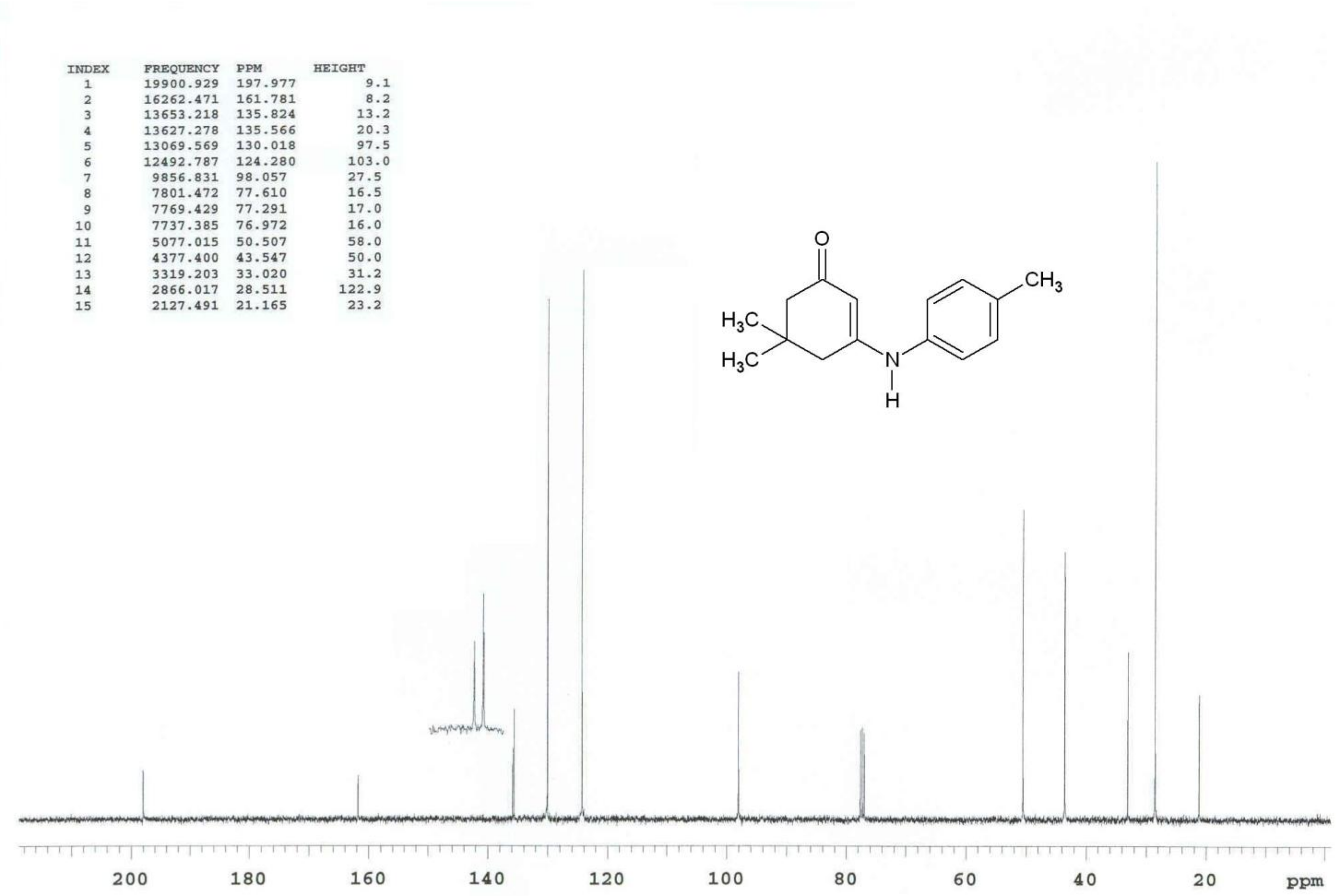

INDEX	FREQUENCY	PPM	HEIGHT
1	19900.929	197.977	9.1
2	16262.471	161.781	8.2
3	13653.218	135.824	13.2
4	13627.278	135.566	20.3
5	13069.569	130.018	97.5
6	12492.787	124.280	103.0
7	9856.831	98.057	27.5
8	7801.472	77.610	16.5
9	7769.429	77.291	17.0
10	7737.385	76.972	16.0
11	5077.015	50.507	58.0
12	4377.400	43.547	50.0
13	3319.203	33.020	31.2
14	2866.017	28.511	122.9
15	2127.491	21.165	23.2

Figure S11. ¹³C NMR spectrum of compound 3c ($CDCl_3$).

Compound 3c - EI-MS

$C_{15}H_{19}NO$

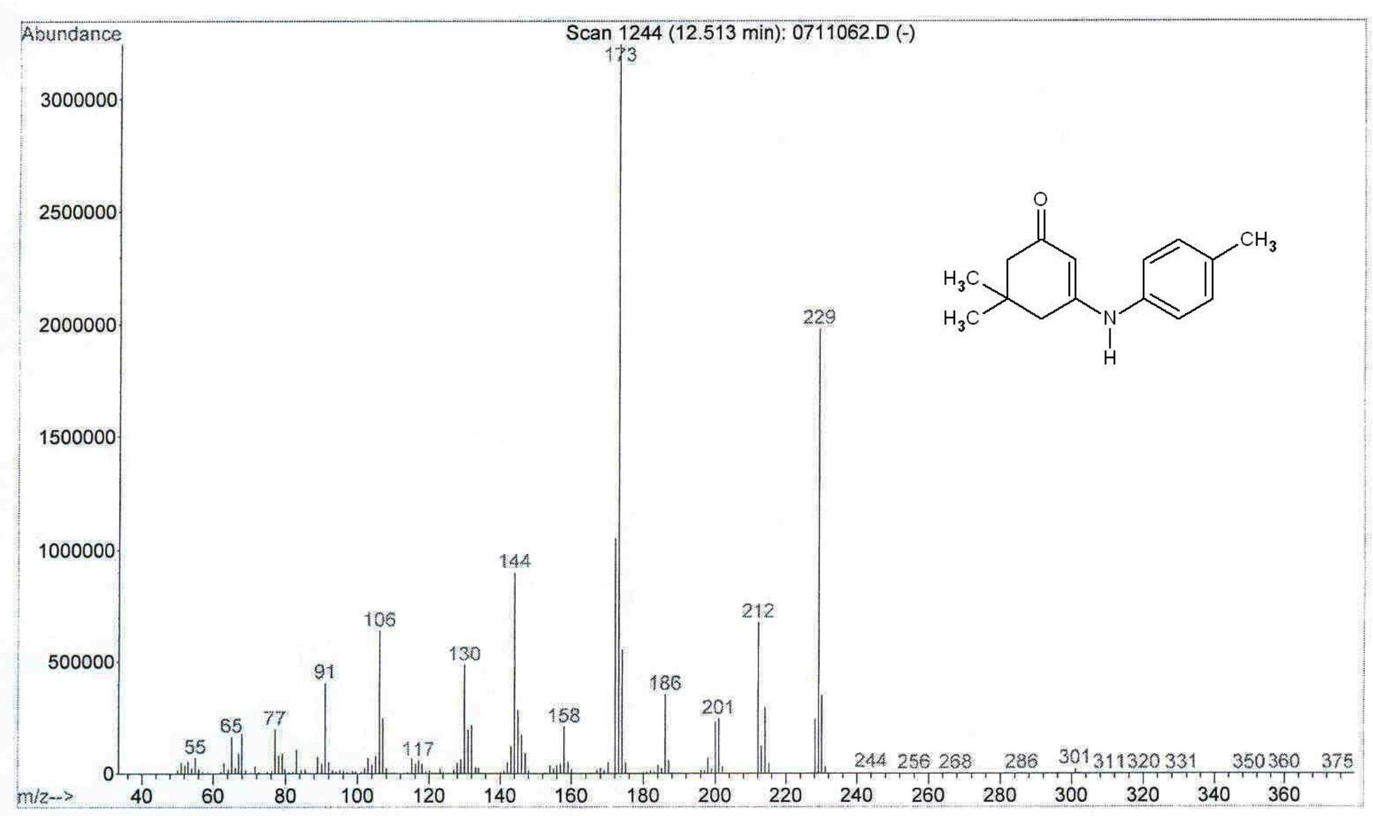

Figure S12. EI mass spectrum of compound 3c.

Compound 5a - IR

9-(2-Thienyl)-10-(4-methoxyphenyl)-3,3,6,6-tetramethyl-1,8-dioxo-1,2,3,4,5,6,7,8,9,10-decahydroacridine

$C_{28}H_{31}NO_3S$

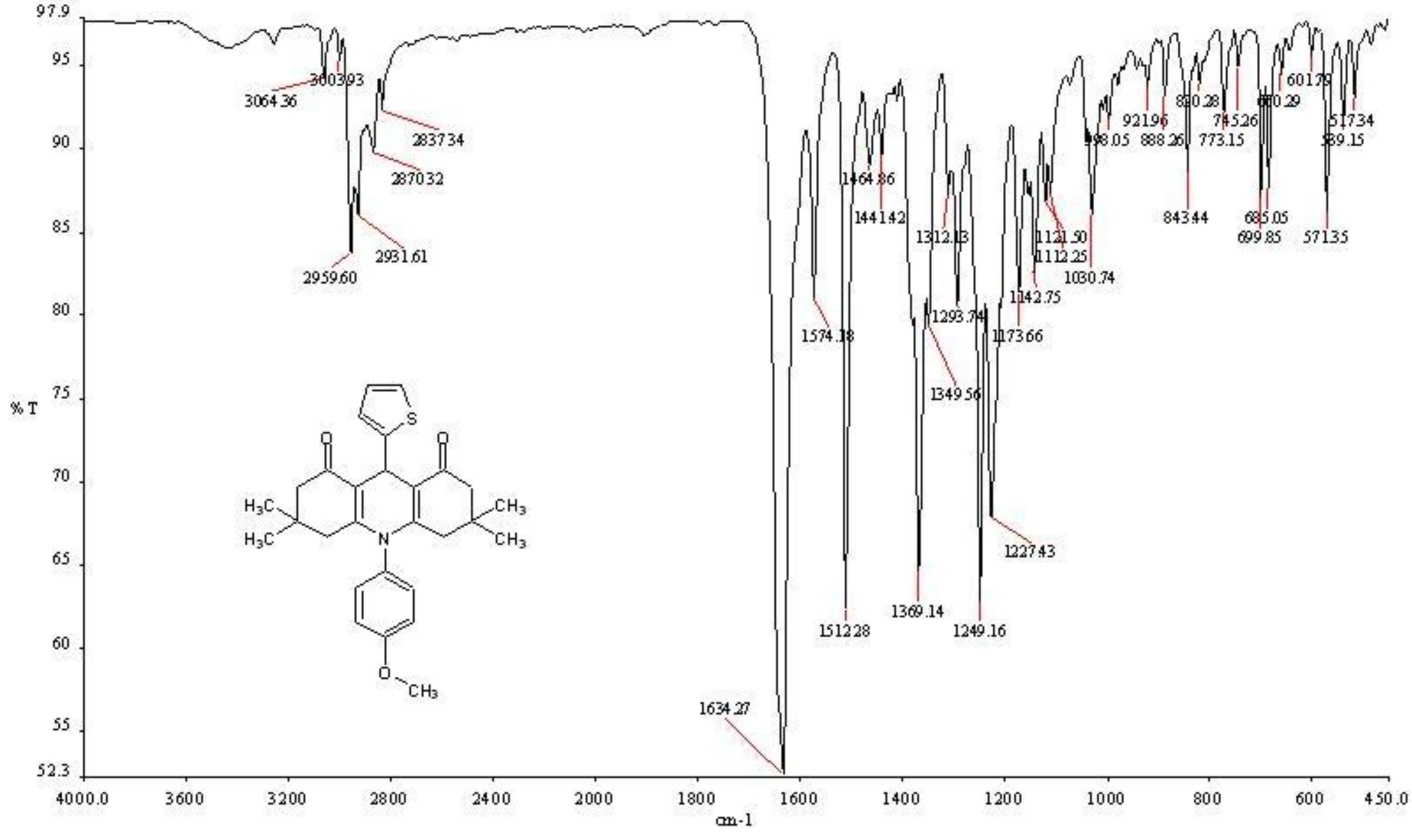

Figure S13. IR spectrum of compound 5a (KBr).

Compound 5a - ¹H NMR

$C_{28}H_{31}NO_3S$

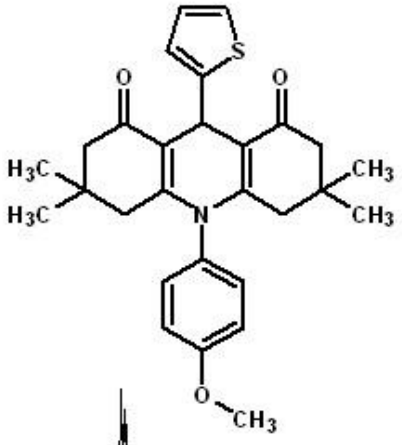

Index	ppm	Hz	Height
1	7.260	2901.956	17.183
2	7.189	2873.750	22.361
3	7.169	2865.890	25.287
4	7.120	2846.264	20.979
5	7.100	2838.309	24.497
6	7.019	2805.796	108.560
7	6.997	2796.970	107.582
8	6.982	2790.842	31.320
9	6.850	2738.377	36.603
10	6.841	2734.833	36.773
11	6.838	2733.424	32.463
12	6.829	2729.840	23.207
13	5.646	2256.979	57.975
14	3.895	1557.081	428.652
15	2.243	896.345	23.904
16	2.202	880.136	213.610
17	2.193	876.699	193.527
18	2.152	860.205	21.219
19	2.104	840.812	82.705
20	2.060	823.283	114.588
21	1.853	740.779	114.317
22	1.810	723.250	81.730
23	0.951	380.032	626.382
24	0.852	340.515	636.903

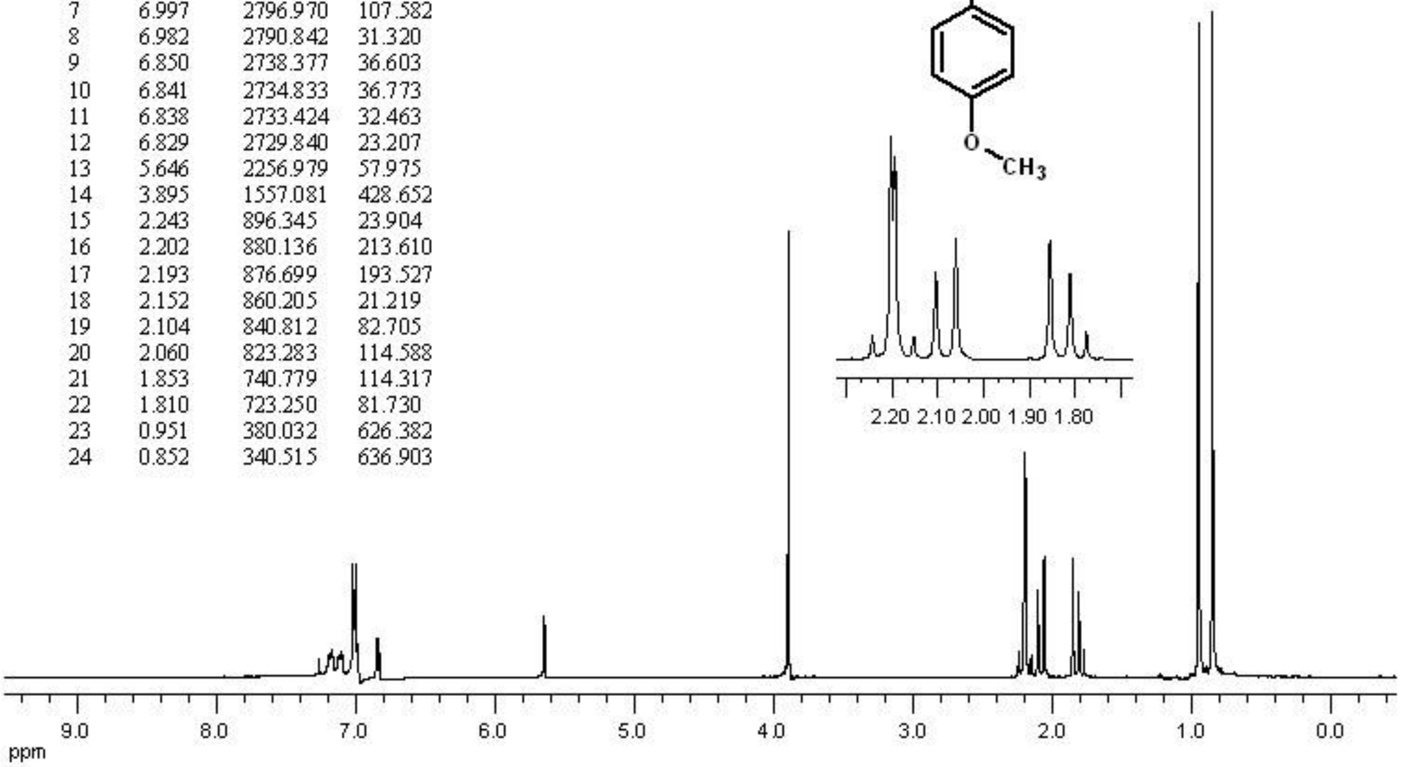

INDEX	FREQUENCY	PPM	HEIGHT	INDEX	FREQUENCY	PPM	HEIGHT
1	2901.693	7.258	3.1	35	402.472	1.007	1.4
2	2873.518	7.188	3.9	36	398.813	0.998	1.4
3	2865.467	7.168	5.0	37	394.056	0.986	1.7
4	2845.708	7.118	3.9	38	380.151	0.951	129.3
5	2838.024	7.099	5.0	39	370.637	0.927	2.2
6	2805.823	7.019	20.6	40	362.953	0.908	1.2
7	2801.066	7.007	11.6	41	359.294	0.899	2.3
8	2796.675	6.996	24.0	42	354.171	0.886	1.8
9	2790.820	6.981	8.4	43	340.632	0.852	130.8
10	2738.128	6.849	7.4	44	323.800	0.810	0.6
11	2734.835	6.841	7.7	45	320.140	0.801	0.9
12	2733.371	6.837	7.5	46	316.481	0.792	1.0
13	2729.712	6.828	5.9	47	276.962	0.693	0.8
14	2256.946	5.646	12.0				
15	1575.606	3.941	0.6				
16	1571.215	3.930	0.7				
17	1564.994	3.915	1.4				
18	1556.944	3.895	88.3				
19	1547.430	3.871	0.7				
20	1530.964	3.830	0.6				
21	896.462	2.242	5.1				
22	879.995	2.201	44.0				
23	876.702	2.193	39.7				
24	860.236	2.152	4.4				
25	840.842	2.103	17.1				
26	823.278	2.059	23.6				
27	758.876	1.898	0.5				
28	740.946	1.853	23.5				
29	731.067	1.829	0.8				
30	723.382	1.810	16.8				
31	708.746	1.773	5.3				
32	490.292	1.226	0.7				
33	441.991	1.106	0.7				
34	408.693	1.022	0.6				

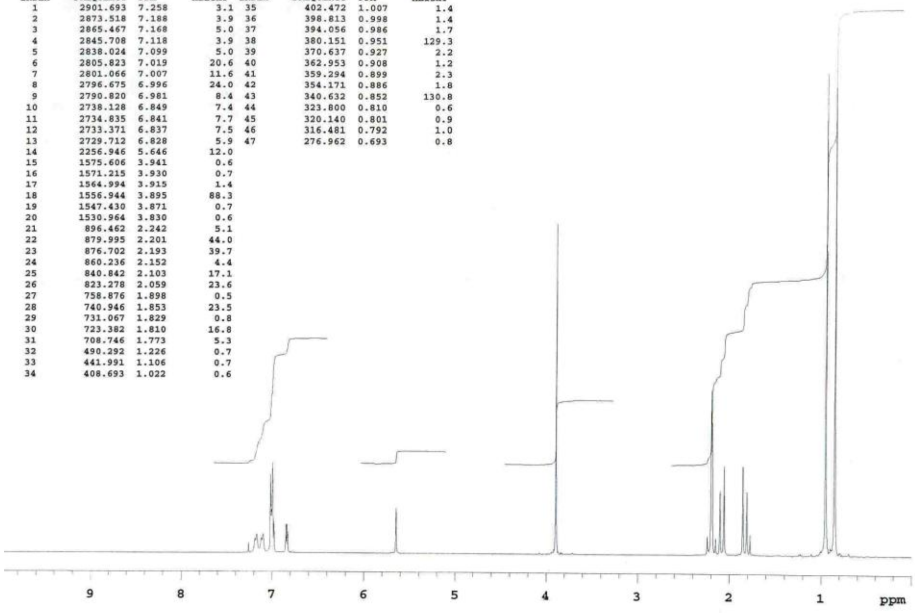

Figure S14. ¹H NMR spectrum of compound 5a (CDCl₃).

Compound 5a - ^{13}C NMR

$C_{28}H_{31}NO_3S$

Index	ppm	Hz	Height
1	195.594	19662.931	1299.423
2	159.776	16062.087	752.061
3	150.524	15131.944	1832.031
4	150.389	15118.375	691.204
5	131.339	13203.301	899.103
6	130.825	13151.676	1257.554
7	130.386	13107.474	1136.918
8	126.878	12754.858	2976.396
9	123.999	12465.377	3372.967
10	122.285	12293.061	3167.661
11	115.758	11636.943	101.290
12	115.250	11585.888	1255.810
13	114.696	11530.179	1124.742
14	114.070	11467.252	1259.037
15	77.318	7772.569	2307.535
16	77.000	7740.502	2350.494
17	76.682	7708.569	2342.996
18	55.551	5584.301	2868.185
19	50.173	5043.617	6534.393
20	41.584	4180.139	7201.397
21	32.270	3243.780	5032.884
22	29.861	3001.629	7073.401
23	27.236	2737.762	2964.036
24	26.709	2684.718	7764.894

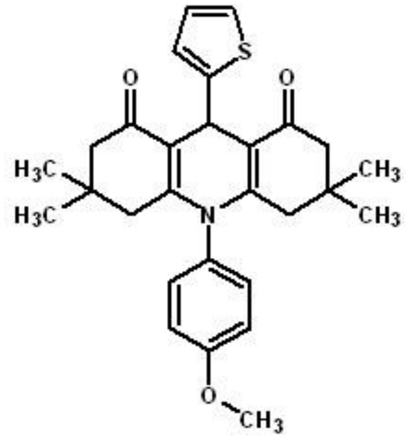

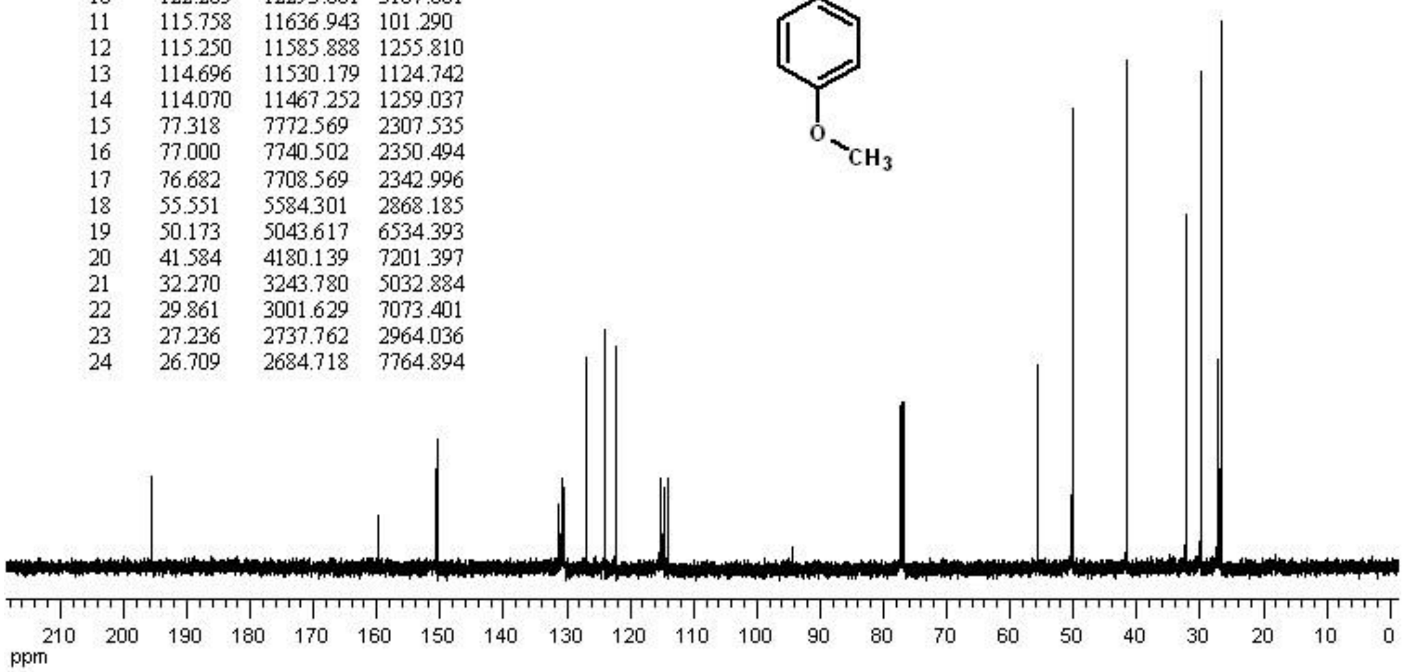

Figure S15. ^{13}C NMR spectrum of compound 5a ($CDCl_3$).

Compound 5a - EI-MS

$C_{28}H_{31}NO_3S$

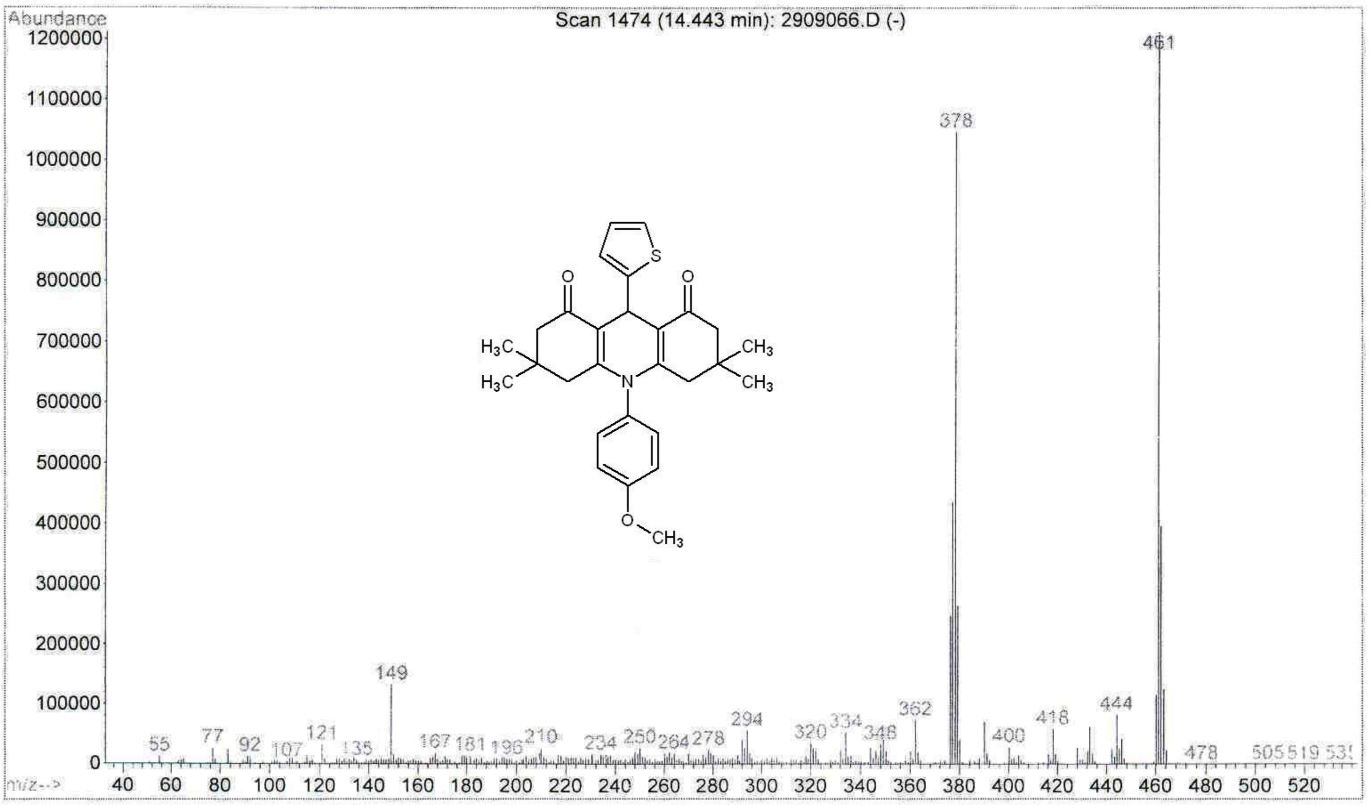

Figure S16. EI mass spectrum of compound 5a.

Compound 5c - IR

9-(2-Thienyl)-10-(4-methylphenyl)-3,3,6,6-tetramethyl-1,8-dioxo-1,2,3,4,5,6,7,8,9,10-decahydroacridine

$C_{28}H_{31}NO_2S$

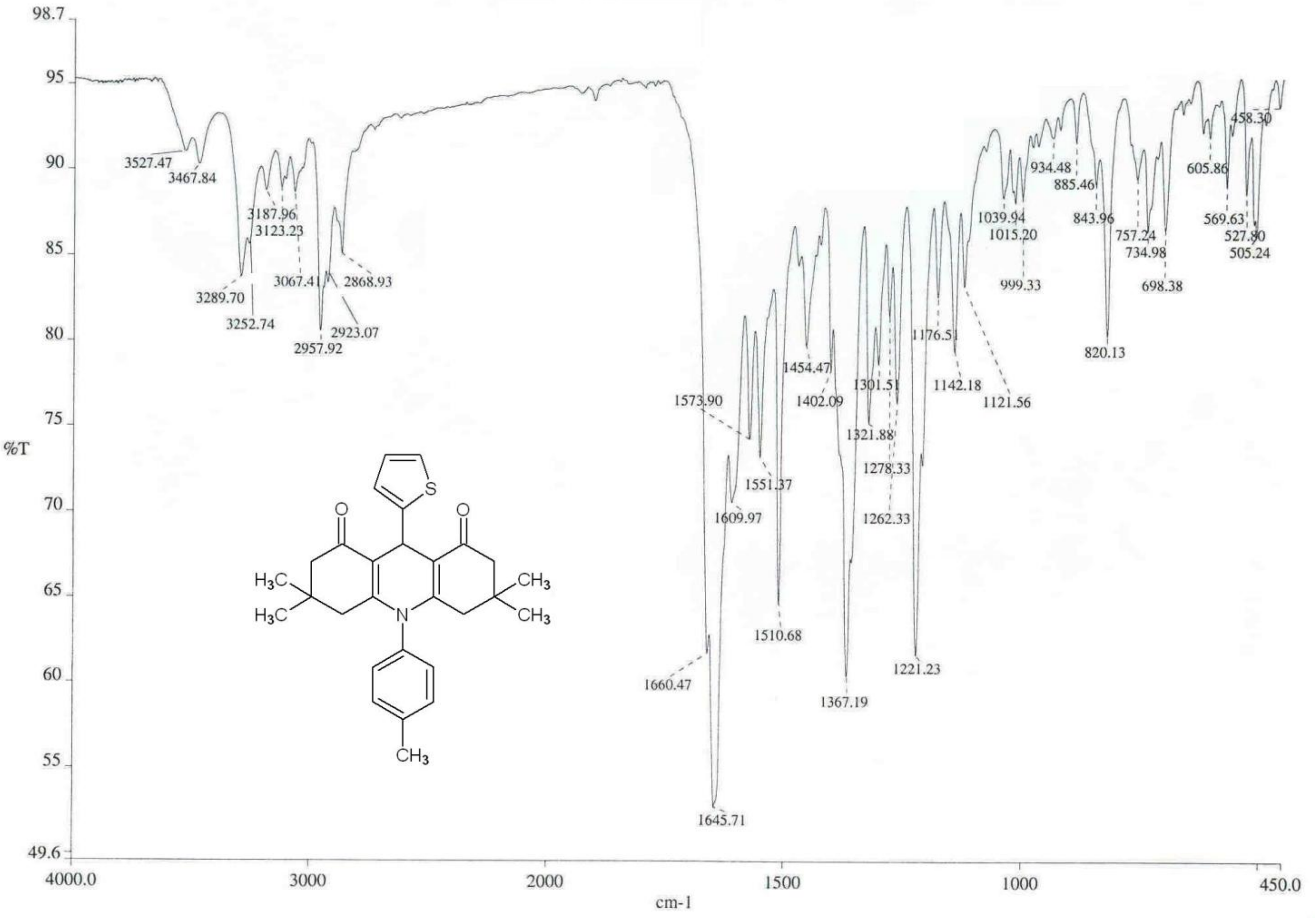

Figure S17. IR spectrum of compound 5c (KBr).

Compound 5c - ¹H NMR

$C_{28}H_{31}NO_2S$

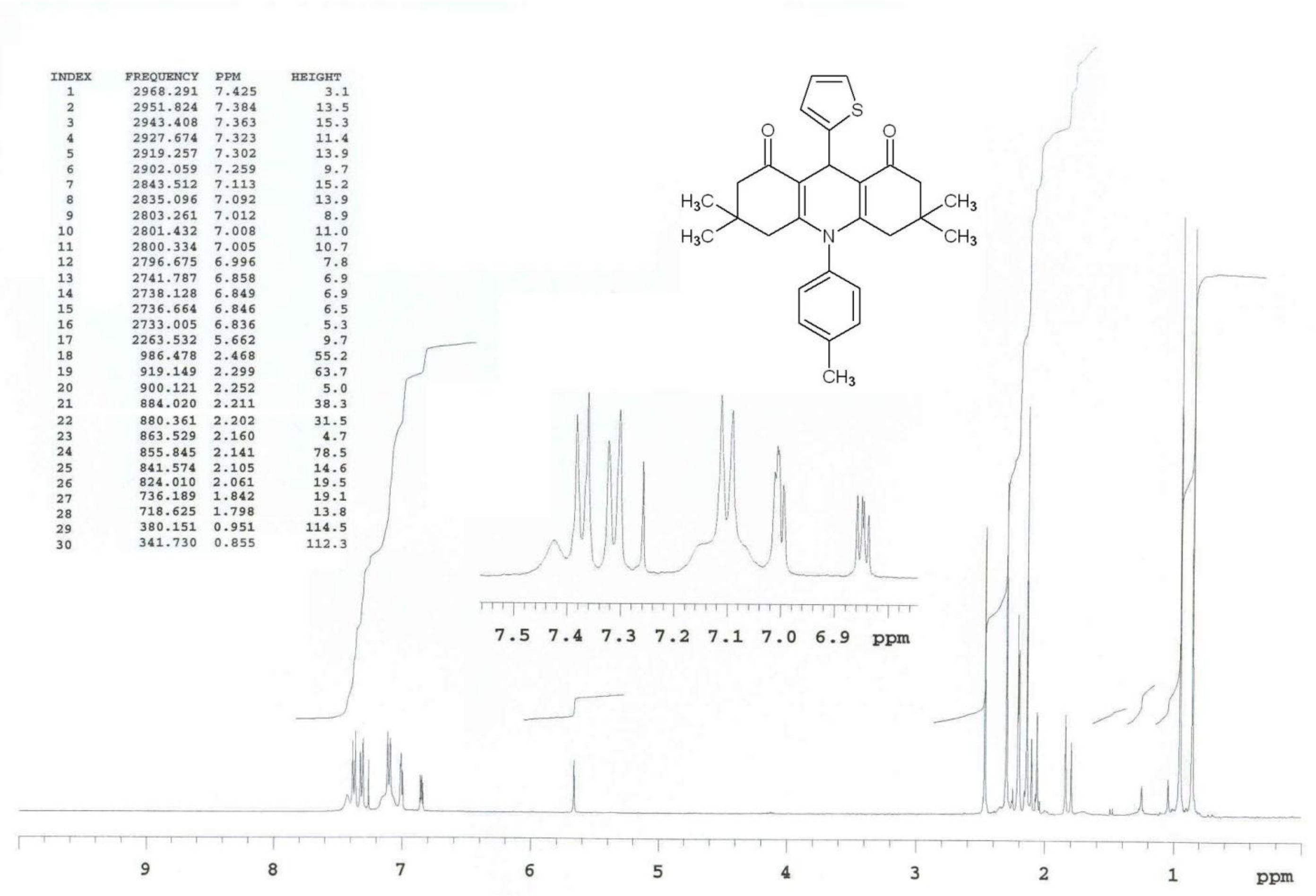

INDEX	FREQUENCY	PPM	HEIGHT
1	2968.291	7.425	3.1
2	2951.824	7.384	13.5
3	2943.408	7.363	15.3
4	2927.674	7.323	11.4
5	2919.257	7.302	13.9
6	2902.059	7.259	9.7
7	2843.512	7.113	15.2
8	2835.096	7.092	13.9
9	2803.261	7.012	8.9
10	2801.432	7.008	11.0
11	2800.334	7.005	10.7
12	2796.675	6.996	7.8
13	2741.787	6.858	6.9
14	2738.128	6.849	6.9
15	2736.664	6.846	6.5
16	2733.005	6.836	5.3
17	2263.532	5.662	9.7
18	986.478	2.468	55.2
19	919.149	2.299	63.7
20	900.121	2.252	5.0
21	884.020	2.211	38.3
22	880.361	2.202	31.5
23	863.529	2.160	4.7
24	855.845	2.141	78.5
25	841.574	2.105	14.6
26	824.010	2.061	19.5
27	736.189	1.842	19.1
28	718.625	1.798	13.8
29	380.151	0.951	114.5
30	341.730	0.855	112.3

Figure S18. ¹H NMR spectrum of compound 5c ($CDCl_3$).

Compound 5c - ^{13}C NMR

$C_{28}H_{31}NO_2S$

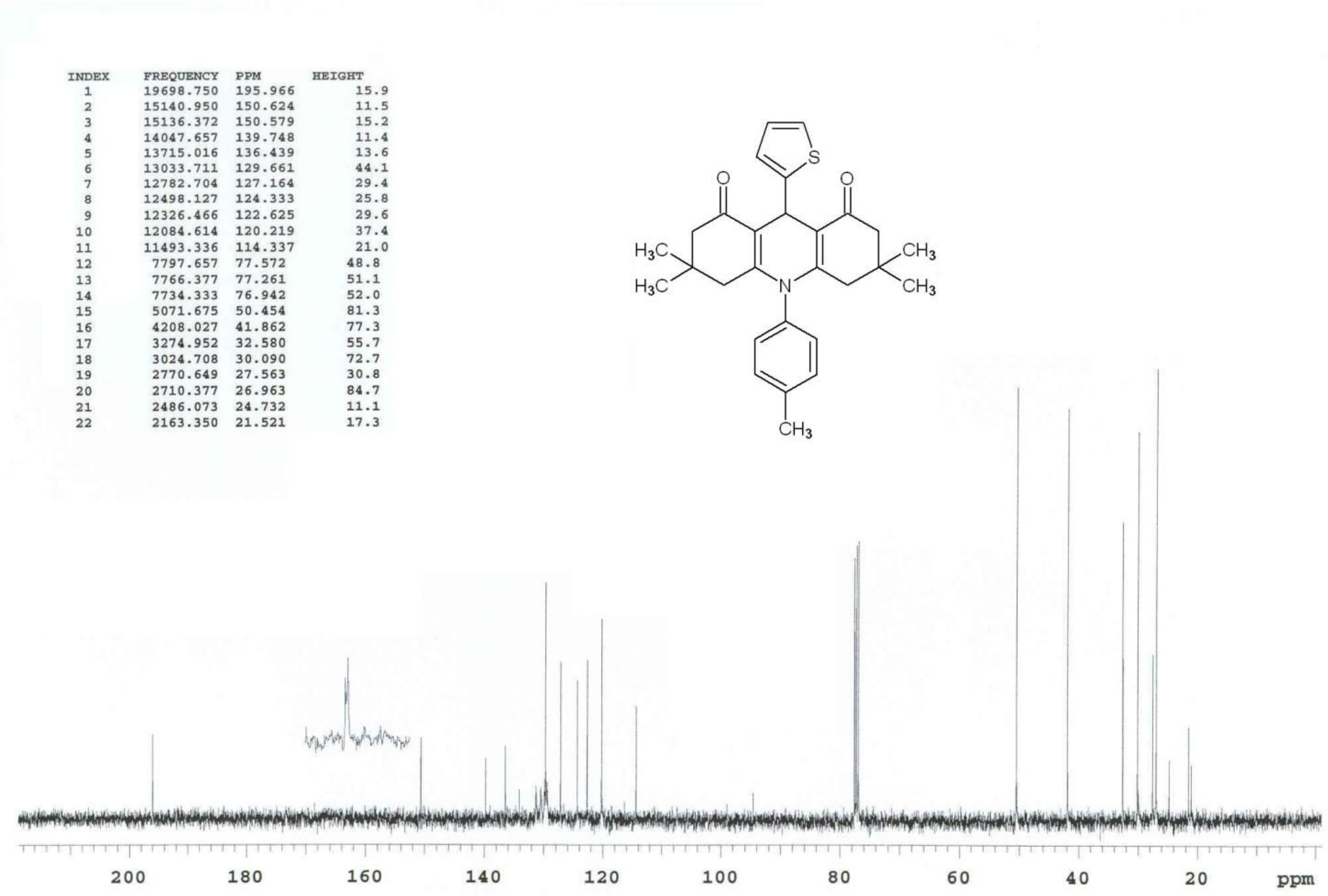

INDEX	FREQUENCY	PPM	HEIGHT
1	19698.750	195.966	15.9
2	15140.950	150.624	11.5
3	15136.372	150.579	15.2
4	14047.657	139.748	11.4
5	13715.016	136.439	13.6
6	13033.711	129.661	44.1
7	12782.704	127.164	29.4
8	12498.127	124.333	25.8
9	12326.466	122.625	29.6
10	12084.614	120.219	37.4
11	11493.336	114.337	21.0
12	7797.657	77.572	48.8
13	7766.377	77.261	51.1
14	7734.333	76.942	52.0
15	5071.675	50.454	81.3
16	4208.027	41.862	77.3
17	3274.952	32.580	55.7
18	3024.708	30.090	72.7
19	2770.649	27.563	30.8
20	2710.377	26.963	84.7
21	2486.073	24.732	11.1
22	2163.350	21.521	17.3

Figure S19. ^{13}C NMR spectrum of compound 5c ($CDCl_3$).

Compound 5c - EI-MS

$C_{28}H_{31}NO_2S$

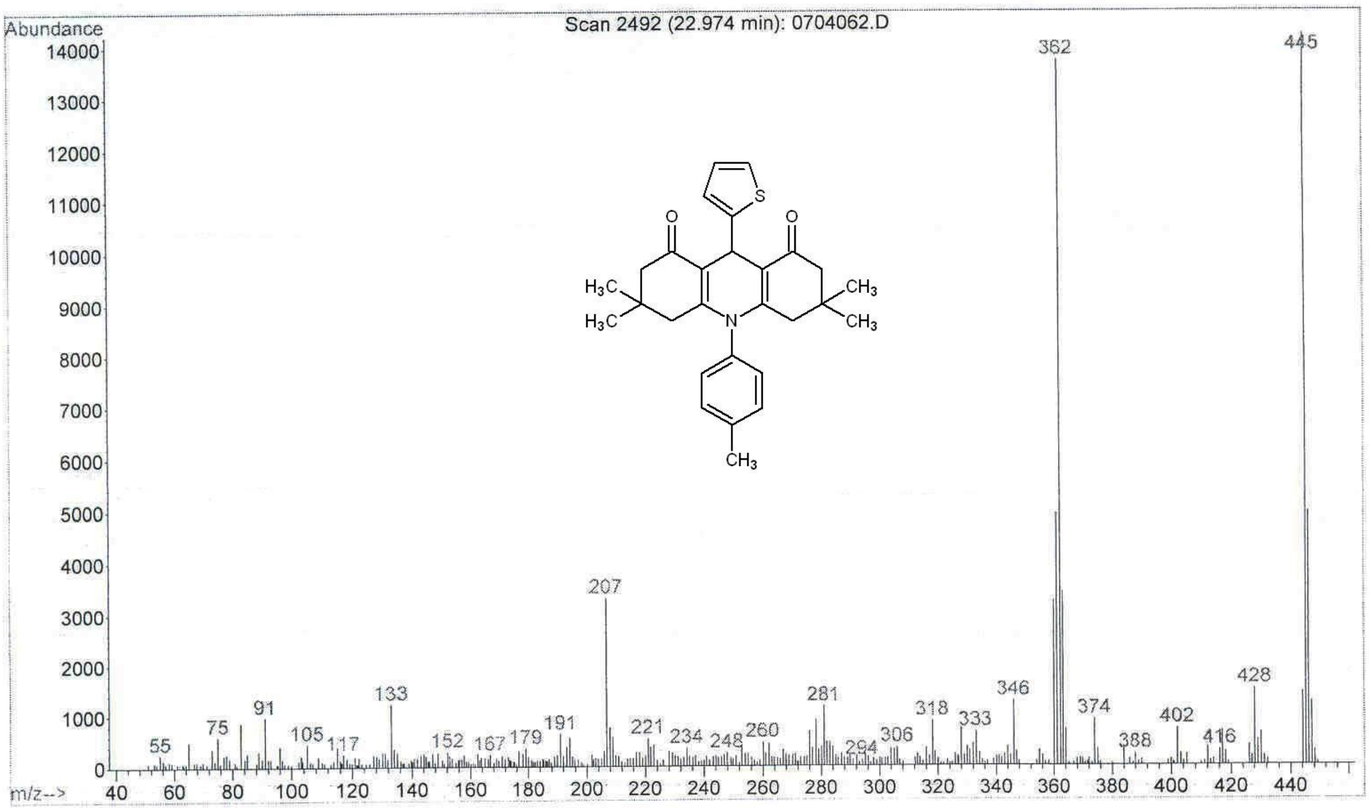

Figure S20. EI mass spectrum of compound 5c.

Compound 5f - IR

9-(3-Methyl-2-thienyl)-10-(4-methylphenyl)-3,3,6,6-tetramethyl-1,8-dioxo-1,2,3,4,5,6,7,8,9,10-decahydroacridine

$C_{29}H_{33}NO_2S$

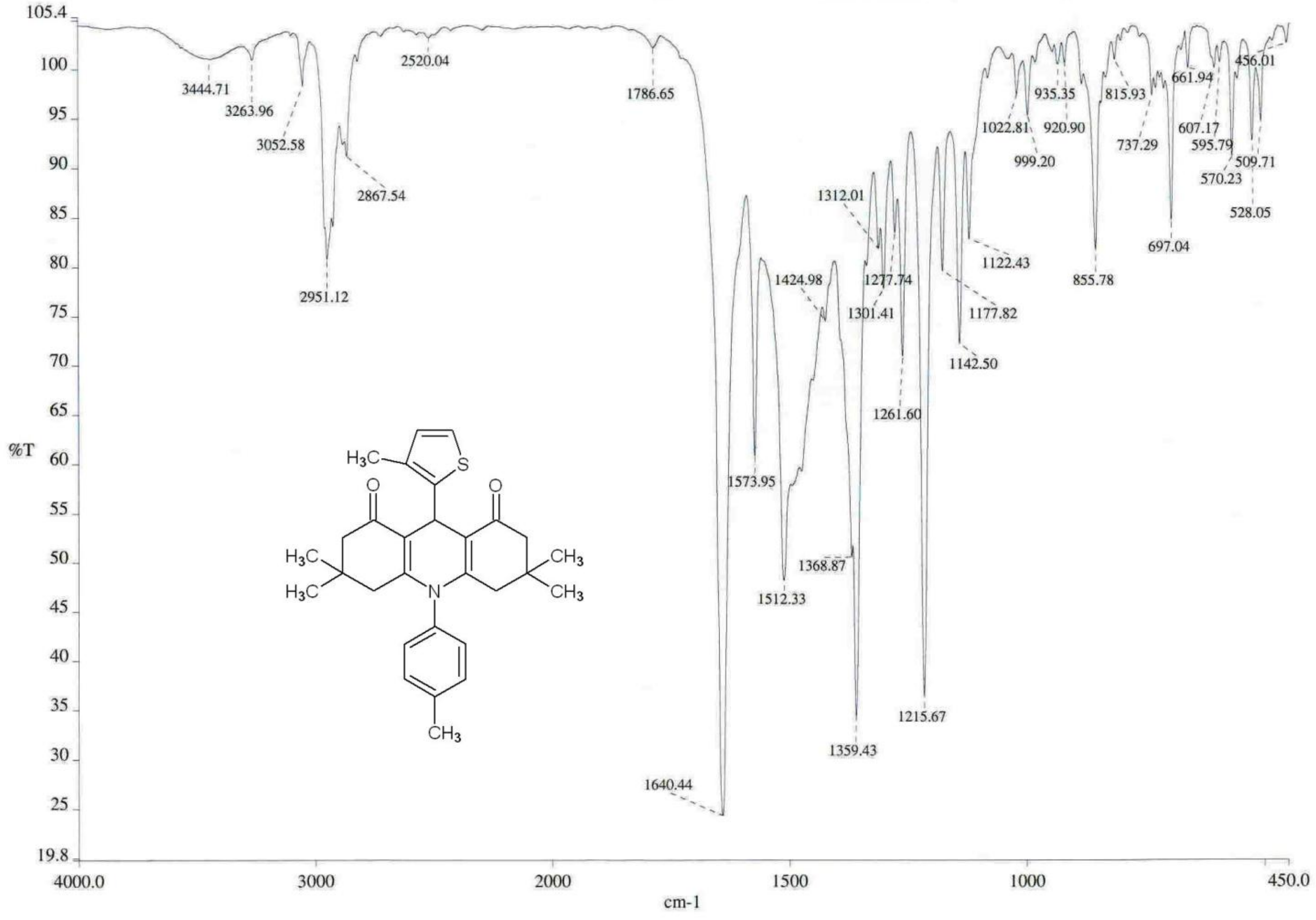

Figure S21. IR spectrum of compound 5f (KBr).

Compound 5f - ^{1}H NMR

$C_{29}H_{33}NO_2S$

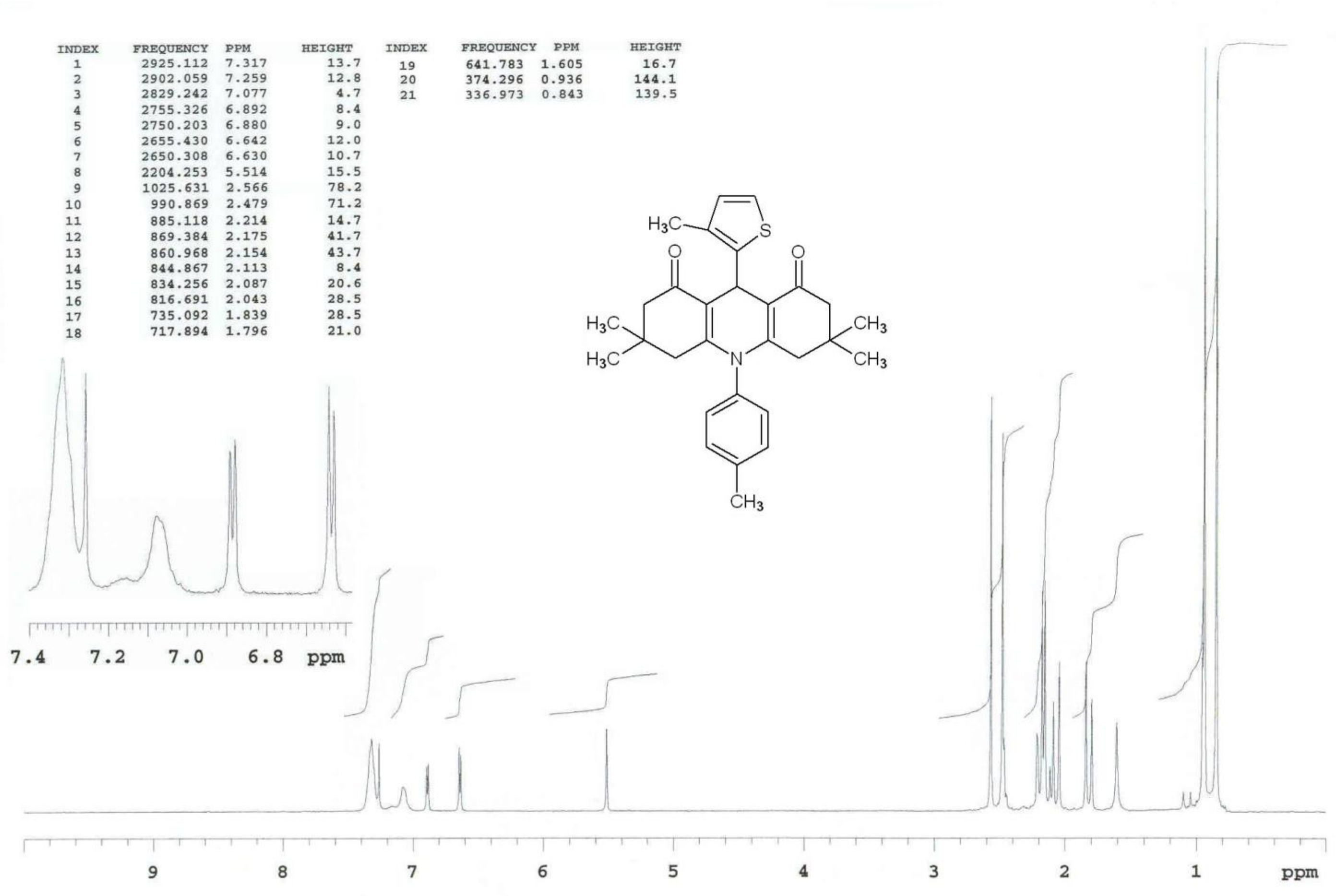

Figure S22. ^{1}H NMR spectrum of compound 5f ($CDCl_3$).

Compound 5f - ^{13}C NMR

$C_{29}H_{33}NO_2S$

INDEX	FREQUENCY	PPM	HEIGHT
1	19692.646	195.905	23.1
2	15066.945	149.888	28.6
3	14035.450	139.626	20.7
4	13743.245	136.720	12.3
5	13447.987	133.782	15.8
6	13113.057	130.450	10.1
7	13077.198	130.094	46.0
8	13050.496	129.828	10.0
9	13015.400	129.479	11.0
10	12136.494	120.735	37.8
11	11597.859	115.377	18.7
12	7796.132	77.557	109.7
13	7764.088	77.238	115.1
14	7732.045	76.919	116.8
15	5070.149	50.438	92.3
16	4212.605	41.908	102.9
17	3274.952	32.580	46.4
18	3026.997	30.113	96.6
19	2702.748	26.887	97.5
20	2656.971	26.432	42.0
21	2164.113	21.529	21.0
22	1437.031	14.296	27.1

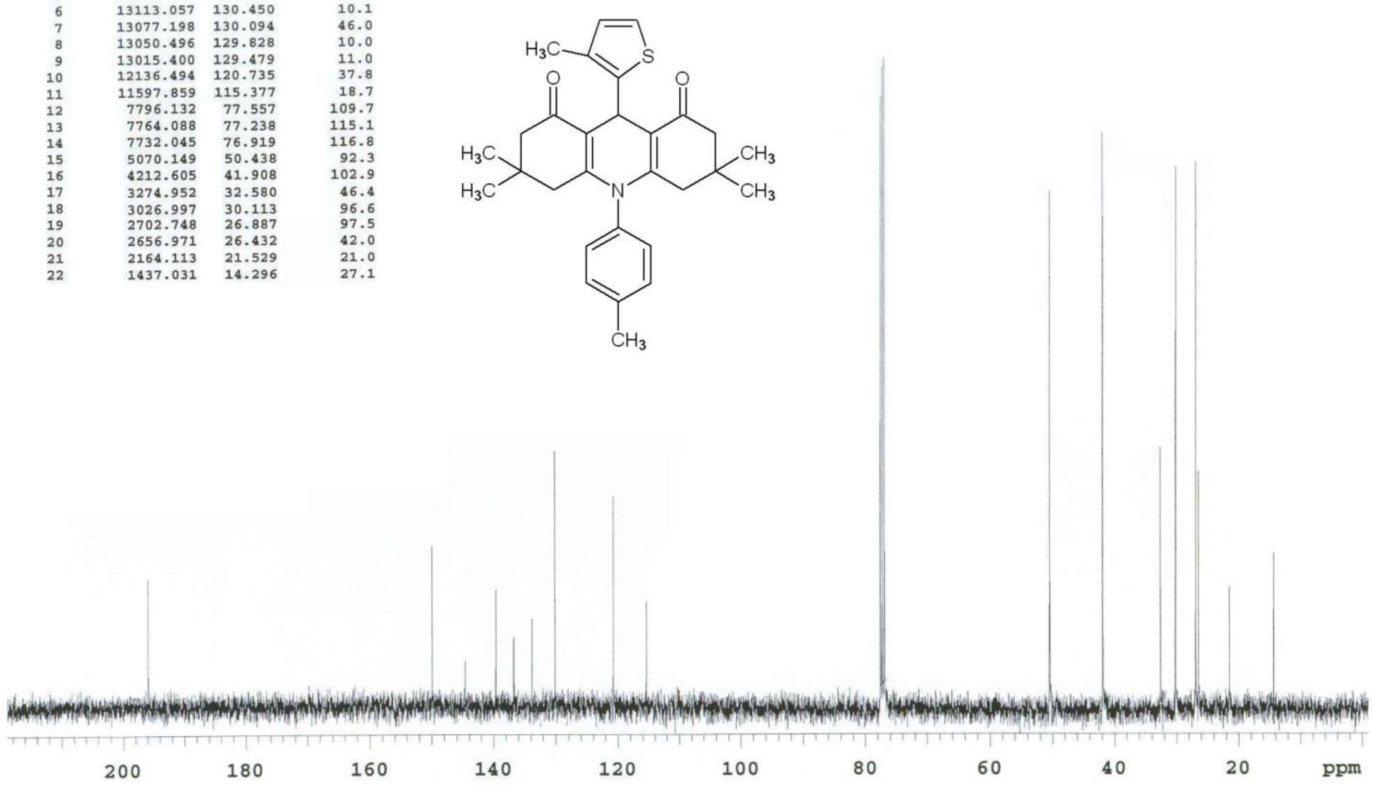

Figure S23. ^{13}C NMR spectrum of compound 5f ($CDCl_3$).

Compound 5f - EI-MS

$C_{29}H_{33}NO_2S$

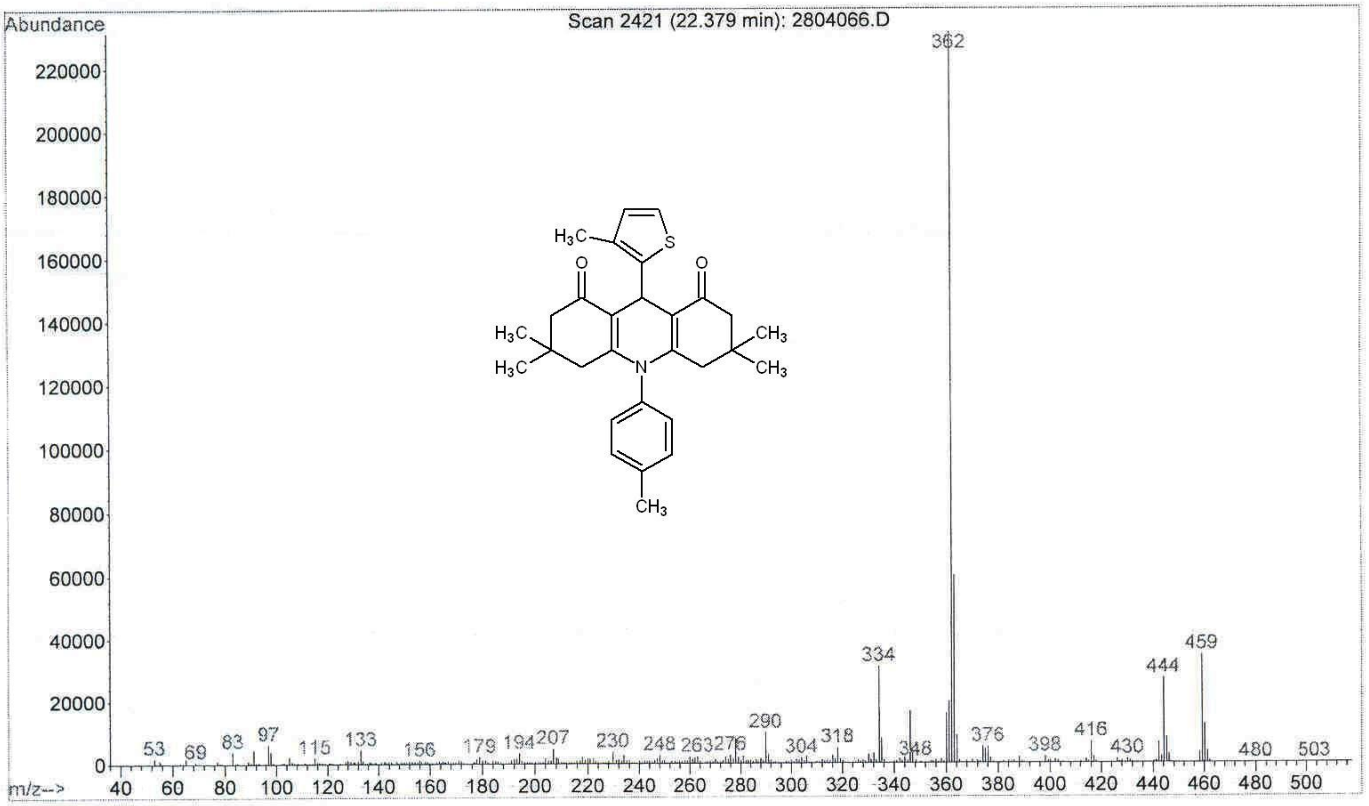

Figure S24. EI mass spectrum of compound 5f.

Compound 6a - IR

3,3-Dimethyl-7-methoxy-1,2,3,4-tetrahydroacridine-1-one

$C_{16}H_{17}NO_2$

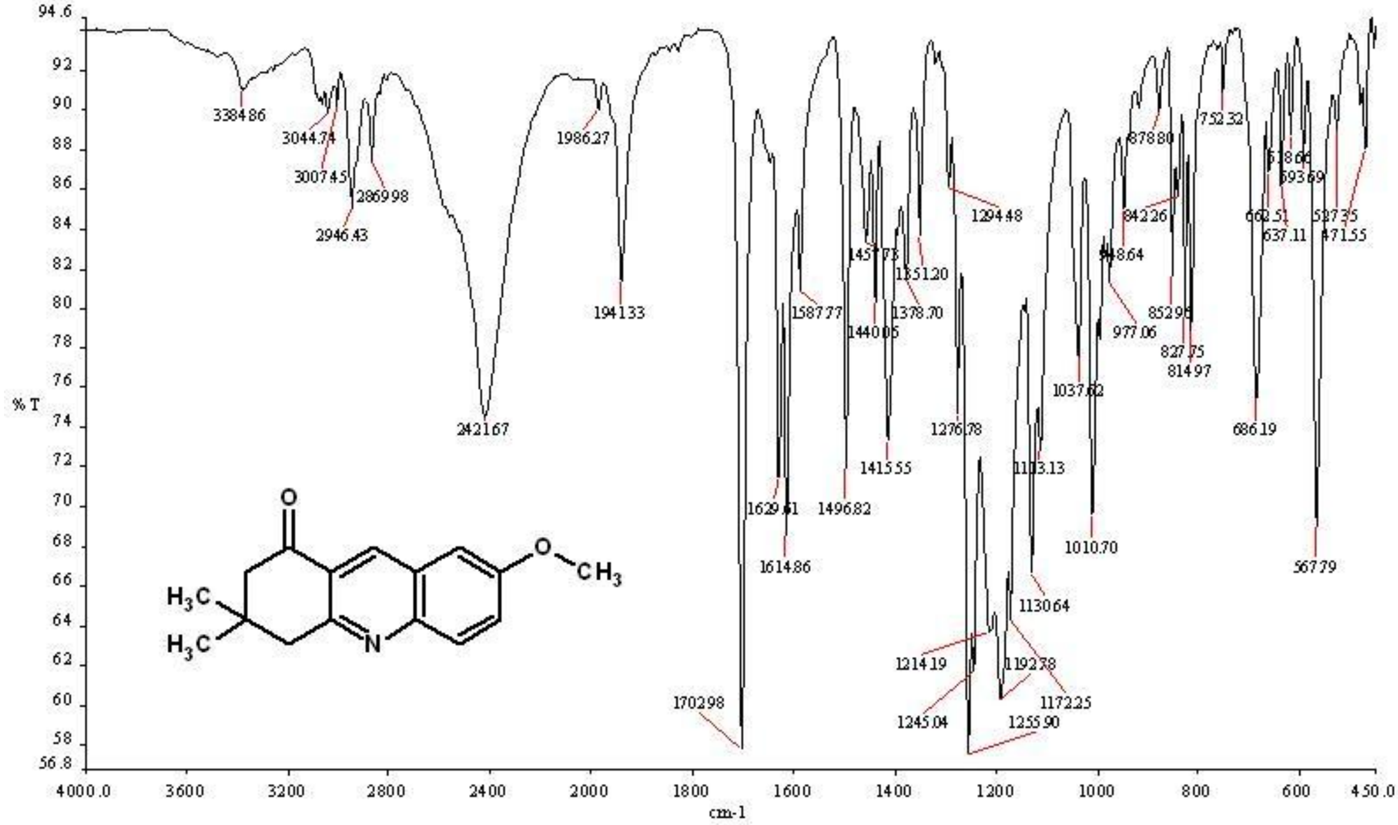

Figure S25. IR spectrum of compound 6a (KBr).

Compound 6a - ¹H NMR

$C_{16}H_{17}NO_2$

Index	ppm	Hz	Height
1	9.188	3672.893	38.811
2	8.632	3450.649	24.981
3	8.608	3441.288	25.216
4	7.863	3143.291	34.436
5	7.843	3135.203	33.769
6	7.696	3076.536	15.217
7	7.689	3073.889	15.432
8	7.672	3067.174	13.875
9	7.666	3064.526	13.687
10	7.387	2953.271	31.630
11	7.381	2950.683	28.229
12	7.260	2902.139	7.494
13	7.192	2875.193	33.616
14	7.172	2867.221	29.278
15	3.982	1591.767	182.487
16	3.521	1407.518	88.359
17	2.679	1070.889	94.902
18	2.347	938.357	140.357
19	1.122	448.581	308.990

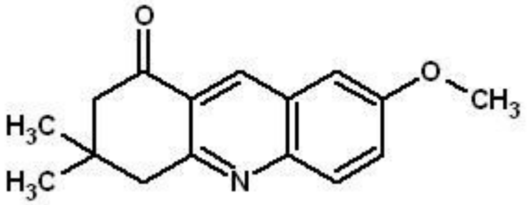

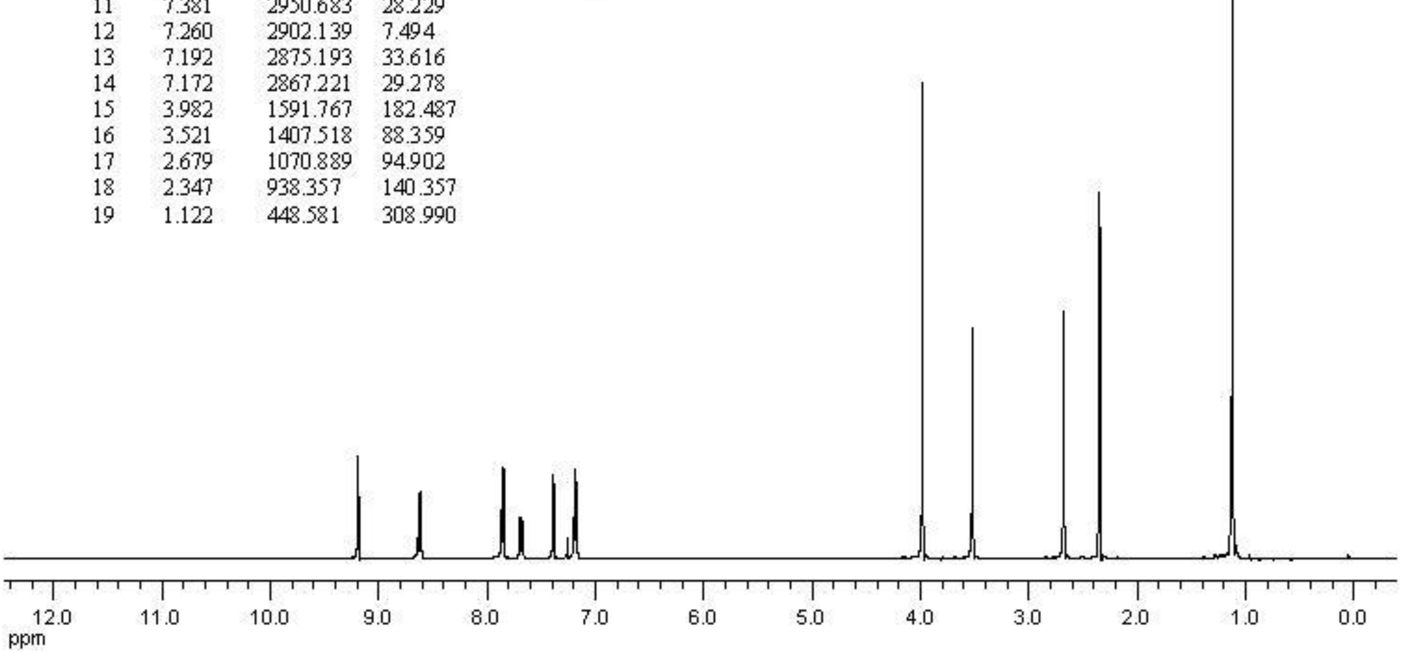

INDEX	FREQUENCY	PPM	HEIGHT
1	3672.683	9.187	17.3
2	3450.571	8.631	11.1
3	3441.057	8.608	11.4
4	3143.199	7.863	15.1
5	3135.149	7.842	15.4
6	3076.602	7.696	6.6
7	3073.675	7.689	6.9
8	3067.088	7.672	6.3
9	3064.527	7.666	6.4
10	2953.288	7.388	13.8
11	2950.726	7.381	12.9
12	2902.059	7.259	3.4
13	2874.981	7.192	14.8
14	2867.297	7.172	13.4
15	1591.706	3.982	81.1
16	1407.283	3.520	38.8
17	1071.005	2.679	41.7
18	938.176	2.347	61.7
19	448.578	1.122	136.9
20	435.405	1.089	2.4

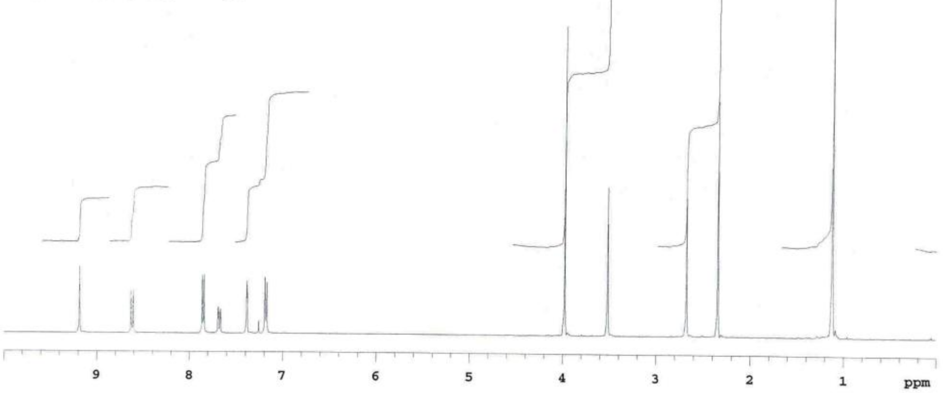

Figure S26. ¹H NMR spectrum of compound 6a ($CDCl_3$).

Compound 6a - ^{13}C NMR

$C_{16}H_{17}NO_2$

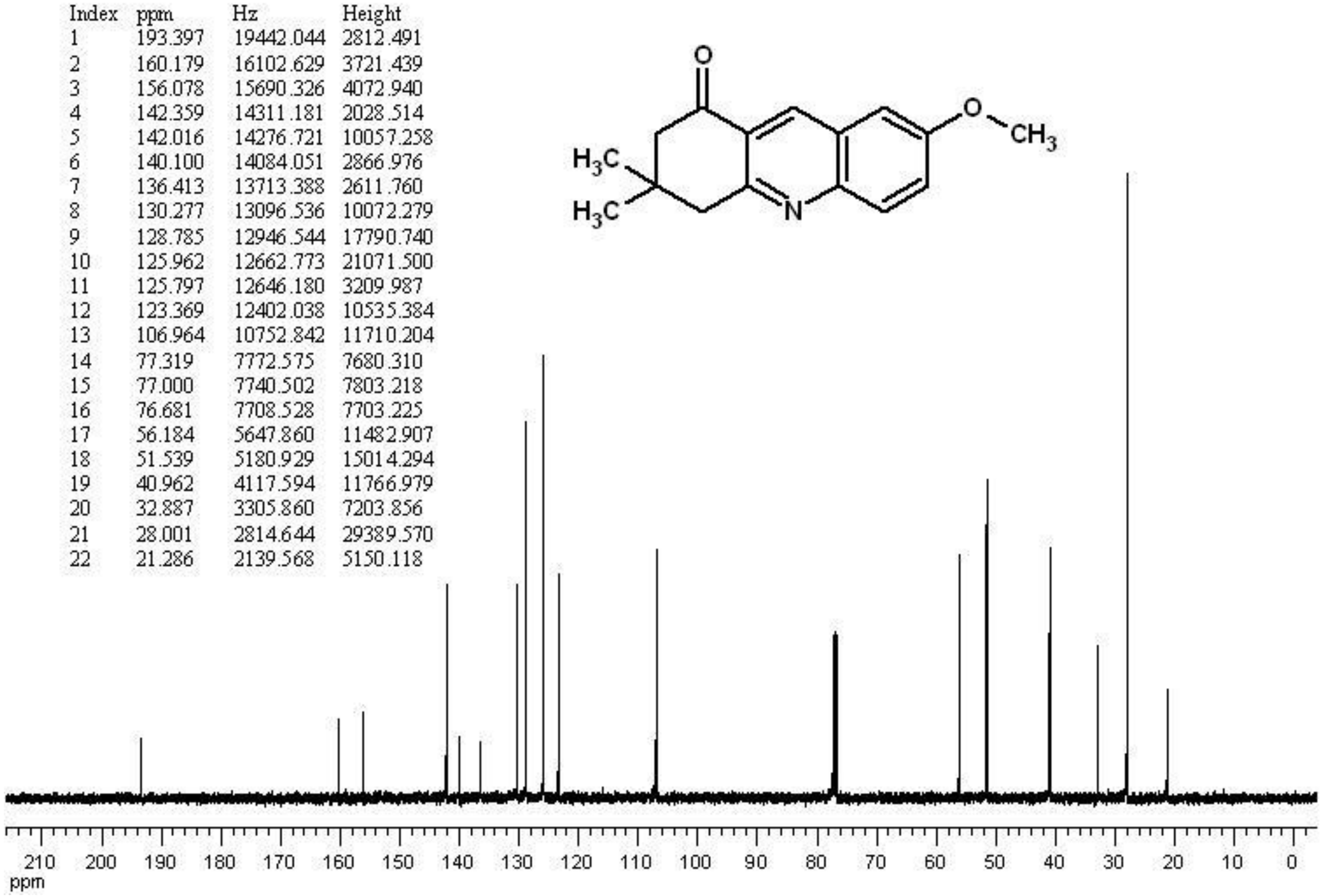

Index	ppm	Hz	Height
1	193.397	19442.044	2812.491
2	160.179	16102.629	3721.439
3	156.078	15690.326	4072.940
4	142.359	14311.181	2028.514
5	142.016	14276.721	10057.258
6	140.100	14084.051	2866.976
7	136.413	13713.388	2611.760
8	130.277	13096.536	10072.279
9	128.785	12946.544	17790.740
10	125.962	12662.773	21071.500
11	125.797	12646.180	3209.987
12	123.369	12402.038	10535.384
13	106.964	10752.842	11710.204
14	77.319	7772.575	7680.310
15	77.000	7740.502	7803.218
16	76.681	7708.528	7703.225
17	56.184	5647.860	11482.907
18	51.539	5180.929	15014.294
19	40.962	4117.594	11766.979
20	32.887	3305.860	7203.856
21	28.001	2814.644	29389.570
22	21.286	2139.568	5150.118

Figure S27. ^{13}C NMR spectrum of compound 6a ($CDCl_3$).

Compound 6a - EI-MS

$C_{16}H_{17}NO_2$

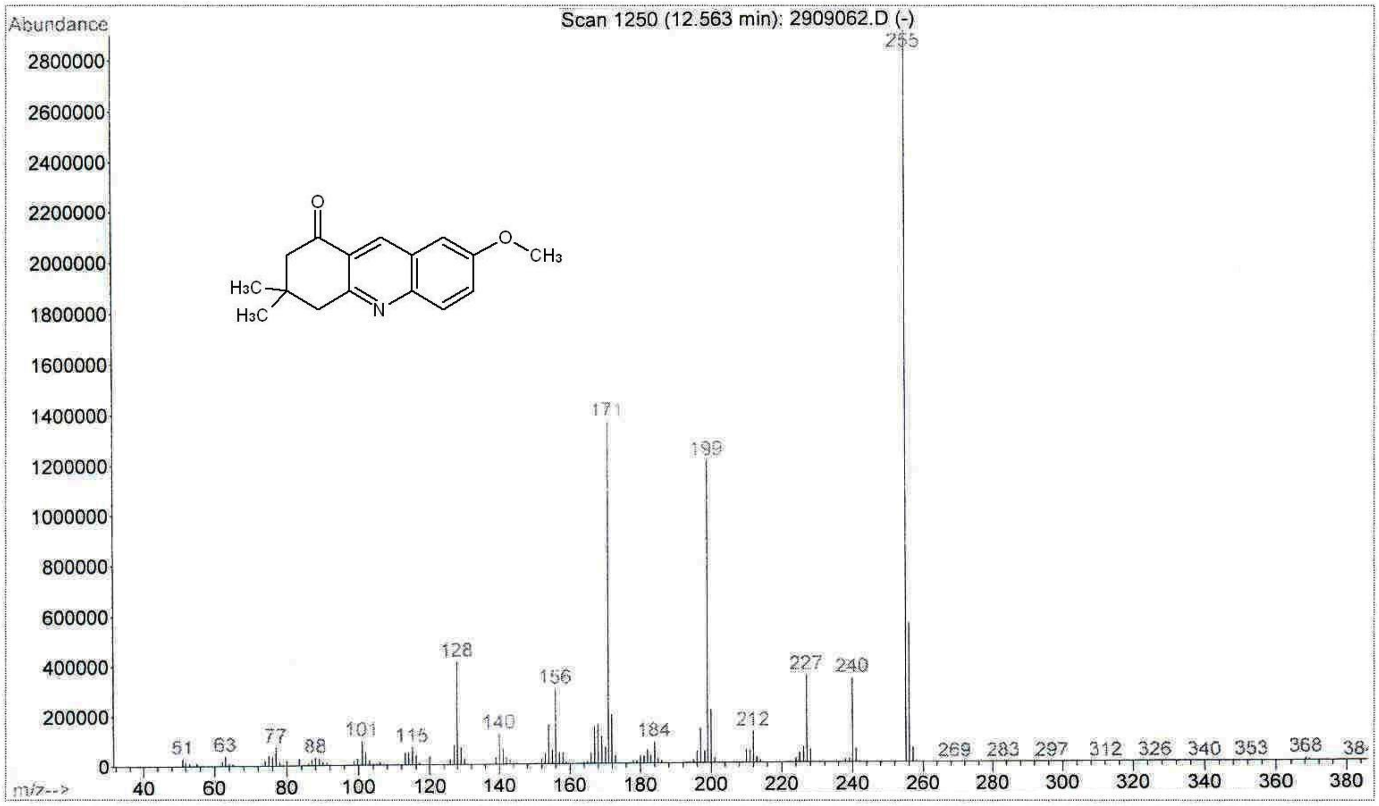

Figure S28. EI mass spectrum of compound 6a.

Historical Numerical Data for Compounds without Surviving Original Printouts

The values in this section were transcribed contemporaneously into the original laboratory records and were not generated from reconstructed spectra. Despite a systematic search, the original spectrum printouts, raw electronic files, and residual samples for these compounds could not be recovered from the surviving post-doctoral and laboratory archives. The numerical records are retained because they contain IR, ^{1}H NMR, ^{13}C NMR, and nominal-mass EI-MS data for each compound; however, they are explicitly distinguished from surviving primary instrument printouts.

Compound 5b

9-(2-Thienyl)-10-(4-ethoxyphenyl)-3,3,6,6-tetramethyl-1,8-dioxo-1,2,3,4,5,6,7,8,9,10-decahydroacridine

$C_{29}H_{33}NO_3S$

Data status: contemporaneously transcribed numerical IR, ^{1}H NMR, ^{13}C NMR, and nominal-mass EI-MS values survive in the original laboratory record; the original printout, raw electronic data, and residual sample could not be recovered from the surviving post-doctoral and laboratory archives. No spectrum has been reconstructed or simulated.

^{1}H-NMR ($CDCl_3$) δ: 0.85 (s, 6H, 2 × CH_3), 0.95 (s, 6H, 2 × CH_3), 1.43 (t, 3H, OCH_2CH_3), 1.83 (d, 2H, 4- and 5-H, $^{2}J_{4,4'}$ = $^{2}J_{5,5'}$ = 17.6 Hz), 2.08 (d, 2H, 4′- and 5′-H), 2.18 (d, 2H, 2- and 7-H, $^{2}J_{2,2'}$ = $^{2}J_{7,7'}$ = 16.5 Hz), 2.22 (d, 2H, 2′- and 7′-H), 4.05 (q, 2H, OCH_2CH_3), 5.65 (s, 1H, 9-H), 6.83–7.18 (m, 7H, hetaryl and aryl); ^{13}C-NMR ($CDCl_3$) δ: 15.02 ($OCH_2\mathbf{C}H_3$), 26.99 (2 × CH_3), 27.52, 30.10, 32.56, 41.85 (C4 and C5), 50.46 (C2 and C7), 63.98 ($O\mathbf{C}H_2CH_3$), 114.36–149.12 (C1a, C8a, hetaryl and aryl), 160.00 (C4a and C5a), 195.77 (C1 and C8); IR (KBr) vmax/cm^{-1}: 3064 (aromatic, =CH), 2959 and 2870 (CH_3), 1634 (C=O), 1574 (C=C), 1512 and 1464 (aromatic, C=C); EIMS *m/z*: 476 [M + 1, 35%], 475 [M+, 100%], 460 [M+ –(CH_3), 5%], 447 [M+ –(CO), 5%], 392 [M+ –(thienyl), 88%].

Compound 5d

9-(3-Methyl-2-thienyl)-10-(4-methoxyphenyl)-3,3,6,6-tetramethyl-1,8-dioxo-1,2,3,4,5,6,7,8,9,10-decahydroacridine

$C_{29}H_{33}NO_3S$

Data status: contemporaneously transcribed numerical IR, ^{1}H NMR, ^{13}C NMR, and nominal-mass EI-MS values survive in the original laboratory record; the original printout, raw electronic data, and residual sample could not be recovered from the surviving post-doctoral and laboratory archives. No spectrum has been reconstructed or simulated.

^{1}H-NMR ($CDCl_3$) δ: 0.85 (s, 6H, 2 × CH_3), 0.94 (s, 6H, 2 × CH_3), 1.82 (d, 2H, 4- and 5-H, $^2J_{4,4'} = {}^2J_{5,5'}$ = 17.4 Hz), 2.07 (d, 2H, 4′- and 5′-H), 2.15 (d, 2H, 2- and 7-H, $^2J_{2,2'} = {}^2J_{7,7'}$ = 15.9 Hz), 2.19 (d, 2H, 2′- and 7′-H), 2.48 (s, 3H, thienyl-CH_3), 3.84 (s, 3H, OCH_3), 5.55 (s, 1H, 9-H), 6.64 (d, 1H, thienyl 4″-H, $^3J_{4'', 5''}$ = 5.1 Hz), 6.89 (d, 1H, thienyl 5″-H), 7.08–7.33 (m, 4H, aryl); ^{13}C-NMR ($CDCl_3$) δ: 14.29 (thienyl-CH_3), 26.92 (2 × CH_3), 30.03, 30.05, 32.58, 41.90 (C4 and C5), 50.46 (C2 and C7), 55.75 (OCH_3), 115.38–145.00 (C1a, C8a, hetaryl and aryl), 149.85 (C4a and C5a), 195.81 (C1 and C8); IR (KBr) νmax/cm^{-1}: 3055 (aromatic, =CH), 2952 and 2865 (CH_3), 1641 (C=O), 1571 (C=C), 1510 and 1422 (aromatic, C=C); EIMS *m/z*: 476 [M + 1, 6%], 475 [M+, 15%], 460 [M+ –(CH_3), 11%], 446 [M+ –(CO), 3%], 378 [M+ –(thienyl-CH_3), 100%].

Compound 5e

9-(3-Methyl-2-thienyl)-10-(4-ethoxyphenyl)-3,3,6,6-tetramethyl-1,8-dioxo-1,2,3,4,5,6,7,8,9,10-decahydroacridine

$C_{30}H_{35}NO_3S$

Data status: contemporaneously transcribed numerical IR, ^{1}H NMR, ^{13}C NMR, and nominal-mass EI-MS values survive in the original laboratory record; the original printout, raw electronic data, and residual sample could not be recovered from the surviving post-doctoral and laboratory archives. No spectrum has been reconstructed or simulated.

^{1}H-NMR ($CDCl_3$) δ: 0.85 (s, 6H, 2 × CH_3), 0.94 (s, 6H, 2 × CH_3), 1.43 (t, 3H, OCH_2CH_3), 1.83 (d, 2H, 4- and 5-H, $^2J_{4,4'}$ = $^2J_{5,5'}$ = 17.4 Hz), 2.07 (d, 2H, 4′- and 5′-H), 2.14 (d, 2H, 2- and 7-H, $^2J_{2,2'}$ = $^2J_{7,7'}$ = 15.9 Hz), 2.18 (d, 2H, 2′- and 7′-H), 2.48 (s, 3H, thienyl-CH_3), 4.05 (q, 2H, OCH_2CH_3), 5.53 (s, 1H, 9-H), 6.64 (d, 1H, thienyl 4″-H, $^3J_{4'', 5''}$ = 5.1 Hz), 6.89 (d, 1H, thienyl 5″-H), 7.08–7.30 (m, 4H, aryl); ^{13}C-NMR ($CDCl_3$) δ: 14.31 (thienyl-CH_3), 15.05 ($OCH_2\mathbf{C}H_3$), 26.90 (2 × CH_3), 30.06, 30.07, 32.58, 41.92 (C4 and C5), 50.44 (C2 and C7), 64.00 ($O\mathbf{C}H_2CH_3$), 115.38–144.05 (C1a, C8a, hetaryl and aryl), 149.82 (C4a and C5a), 195.85 (C1 and C8); IR (KBr) νmax/cm^{-1}: 3053 (aromatic, =CH), 2950 and 2866 (CH_3), 1640 (C=O), 1572 (C=C), 1511 and 1423 (aromatic, C=C); EIMS *m/z*: 490 [M + 1, 5%], 489 [M+, 15%], 474 [M+ –(CH_3), 11%], 460 [M+ –(CO), 3%], 392 [M+ –(thienyl-CH_3), 100%].

Compound 6b

3,3-Dimethyl-7-ethoxy-1,2,3,4-tetrahydroacridine-1-one

$C_{17}H_{19}NO_2$

Data status: contemporaneously transcribed numerical IR, ^{1}H NMR, ^{13}C NMR, and nominal-mass EI-MS values survive in the original laboratory record; the original printout, raw electronic data, and residual sample could not be recovered from the surviving post-doctoral and laboratory archives. No spectrum has been reconstructed or simulated.

^{1}H-NMR ($CDCl_3$) δ: 1.10 (s, 6H, 2 × CH_3), 1.43 (t, 3H, OCH_2CH_3), 2.58 (s, 2H, 4-H), 3.50 (s, 2H, 2-H), 4.05 (q, 2H, OCH_2CH_3), 7.36 (d, 1H, 8-H, $^4J_{6,8}$ = 2.7 Hz), 7.66 (dd, 1H, 6-H, $^3J_{5,6}$ = 9.4 Hz, $^4J_{6,8}$ = 2.7 Hz), 8.60 (d, 1H, 5-H, $^3J_{5,6}$ = 9.4 Hz), 9.21 (s, 1H, 9-H); ^{13}C-NMR ($CDCl_3$) δ: 15.03 (OCH$_2$**C**H$_3$), 28.20 (2 × CH_3), 33.20 (C3), 41.23 (C4), 51.81 (C2), 64.00 (O**C**H$_2$CH$_3$), 107.22 (C8), 123.75 (C6), 130.51 (C5), 136.70 (C8-**C**-C9), 141.40 (C1-**C**-C9), 142.35 (C9), 142.56 (C5-**C**-N), 156.40 (C7), 160.52 (C4-**C**-N), 192.75 (C1); IR (KBr) νmax/cm^{-1}: 3042 (aromatic, =CH), 2946 and 2866 (CH_3), 1702 (C=O), 1630 (C=N), 1611, 1577 and 1495 (aromatic, C=C); EIMS *m/z*: 270 [M + 1, 18%], 269 [M+, 100%], 254 [M+ – (CH_3), 11%], 241 [M+ –(CO), 13%].

Compound 6c

3,3-Dimethyl-7-methyl-1,2,3,4-tetrahydroacridine-1-one

$C_{16}H_{17}NO$

Data status: contemporaneously transcribed numerical IR, ¹H NMR, ¹³C NMR, and nominal-mass EI-MS values survive in the original laboratory record; the original printout, raw electronic data, and residual sample could not be recovered from the surviving post-doctoral and laboratory archives. No spectrum has been reconstructed or simulated.

^{1}H-NMR ($CDCl_3$) δ: 1.08 (s, 6H, 2 × CH_3), 2.43 (s, 2H, 4-H), 2.57 (s, 3H, *7*-CH_3), 3.45 (s, 2H, 2-H), 7.35 (d, 1H, 8-H, $^4J_{6,8}$ = 2.7 Hz), 7.67 (dd, 1H, 6-H, $^3J_{5,6}$ = 9.4 Hz, $^4J_{6,8}$ = 2.7 Hz), 8.63 (d, 1H, 5-H, $^3J_{5,6}$ = 9.4 Hz), 9.17 (s, 1H, 9-H); ^{13}C-NMR ($CDCl_3$) δ: 21.53 (*7*-CH_3), 28.25 (2 × CH_3), 33.17 (C3), 41.22 (C4), 51.85 (C2), 107.27 (C8), 123.65 (C6), 130.62 (C5), 136.77 (C8-**C**-C9), 140.10 (C1-**C**-C9), 142.41 (C9), 142.63 (C5-**C**-N), 156.37 (C7), 160.47 (C4-**C**-N), 193.50 (C1); IR (KBr) νmax/cm^{-1}: 3042 (aromatic, =CH), 2945 and 2868 (CH_3), 1702 (C=O), 1629 (C=N), 1612, 1585 and 1493 (aromatic, C=C); EIMS *m/z*: 240 [M + 1, 18%], 239 [M+, 100%], 224 [M+ –(CH_3), 12%], 211 [M+ –(CO), 12%]. The δ 2.57 ppm resonance is assigned to 7-CH_3, the arylamine-derived ring methyl; an intact p-tolyl or thienyl-methyl substituent is not present in isolated **6c**.

Proposed Xanthene-Type Species B

9-(2-Thienyl)-3,3,6,6-tetramethyl-1,2,3,4,5,6,7,8,9,10-decahydroxanthene-1,8-dione

Interpretive status: detected only as a trace nominal-mass EI-MS feature in archived reaction-mixture data; not isolated as a pure compound. No crude-reaction ¹H NMR spectrum or diagnostic xanthene-type methine signal could be recovered from the surviving post-doctoral and laboratory archives. Constitutional isomers or co-eluting species cannot be excluded, and the structural assignment remains tentative.

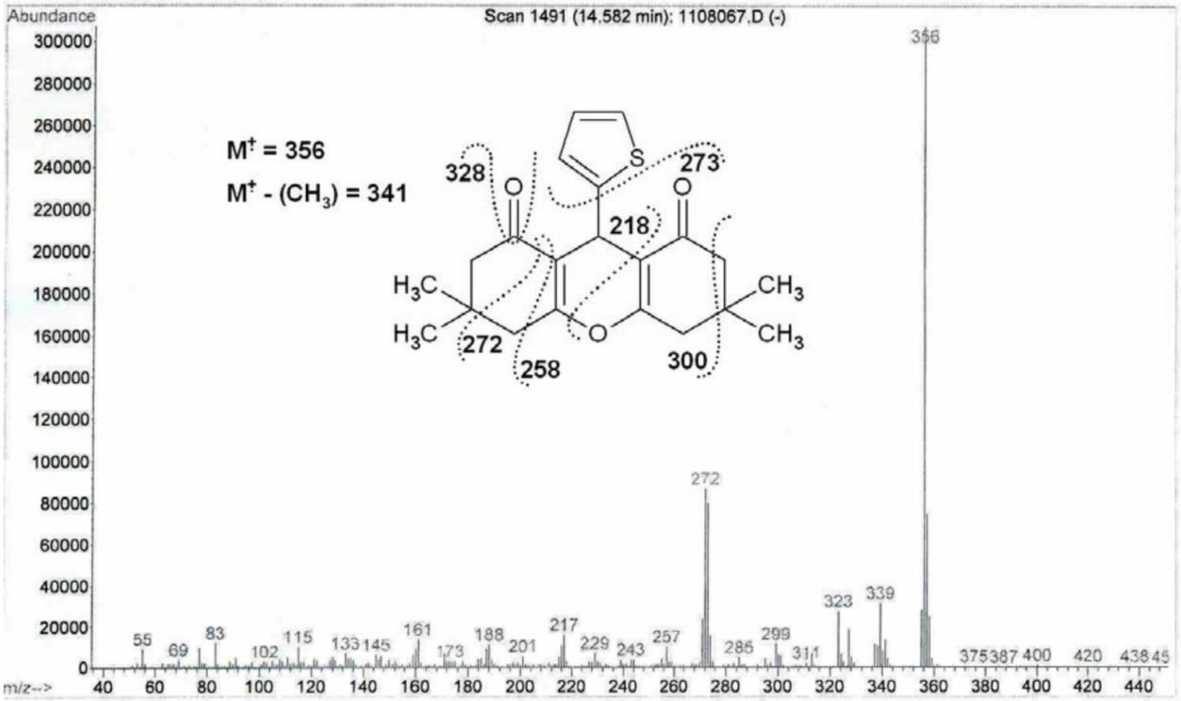

Figure S29. EI-MS spectrum and proposed fragmentation of the xanthene-type species B.

Proposed Tetrahydroacridinone Species D

9-(2-Thienyl)-3,3,7-trimethyl-1,2,3,4-tetrahydroacridin-1-one

Interpretive status: detected only as a trace nominal-mass EI-MS feature in archived reaction-mixture data; not isolated as a pure compound and not confirmed by multidimensional NMR or independent synthesis. Constitutional isomers or co-eluting species cannot be excluded, and the structural assignment remains tentative.

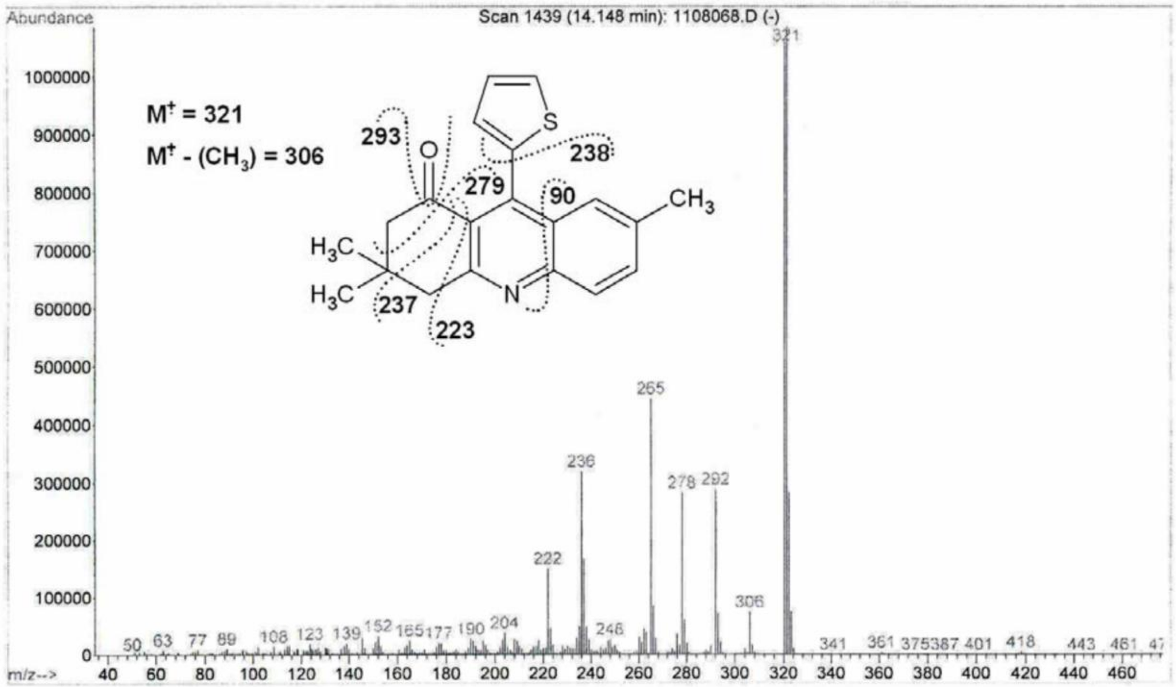

Figure S30. EI-MS spectrum and proposed fragmentation of the tetrahydroacridinone species D.
